# A sequence-based approach for prediction of CsrA/RsmA targets in bacteria with experimental validation in *Pseudomonas aeruginosa*

**DOI:** 10.1093/nar/gku309

**Published:** 2014-04-29

**Authors:** Prajna R. Kulkarni, Tao Jia, Sarah A. Kuehne, Thomas M. Kerkering, Elizabeth R. Morris, Mark S. Searle, Stephan Heeb, Jayasimha Rao, Rahul V. Kulkarni

**Affiliations:** 1 Department of Physics, University of Massachusetts Boston, Boston, MA 02125, USA; 2 Social Cognitive Networks Academic Research Center, and Department of Computer Science, Rensselaer Polytechnic Institute, Troy, NY 12180, USA; 3 School of Life Sciences, Centre for Biomolecular Sciences, University Park, University of Nottingham, Nottingham NG7 2RD, UK; 4 Section of Infectious Diseases, Carilion Clinic/Virginia Tech Carilion School of Medicine/Jefferson College of Health Sciences, Roanoke, VA 24013, USA; 5 School of Chemistry, Centre for Biomolecular Sciences, University Park, University of Nottingham, Nottingham NG7 2RD, UK

## Abstract

CsrA/RsmA homologs are an extensive family of ribonucleic acid (RNA)-binding proteins that function as global post-transcriptional regulators controlling important cellular processes such as secondary metabolism, motility, biofilm formation and the production and secretion of virulence factors in diverse bacterial species. While direct messenger RNA binding by CsrA/RsmA has been studied in detail for some genes, it is anticipated that there are numerous additional, as yet undiscovered, direct targets that mediate its global regulation. To assist in the discovery of these targets, we propose a sequence-based approach to predict genes directly regulated by these regulators. In this work, we develop a computer code (CSRA_TARGET) implementing this approach, which leads to predictions for several novel targets in *Escherichia coli* and *Pseudomonas aeruginosa*. The predicted targets in other bacteria, specifically *Salmonella enterica* serovar Typhimurium*, Pectobacterium carotovorum* and *Legionella pneumophila*, also include global regulators that control virulence in these pathogens, unraveling intricate indirect regulatory roles for CsrA/RsmA. We have experimentally validated four predicted RsmA targets in *P. aeruginosa*. The sequence-based approach developed in this work can thus lead to several testable predictions for direct targets of CsrA homologs, thereby complementing and accelerating efforts to unravel global regulation by this important family of proteins.

## INTRODUCTION

### Background

Successful bacterial persistence and dissemination is critically dependent on global regulatory networks that coordinate cellular functions in response to environmental fluctuations. The extensive family of ribonucleic acid (RNA)-binding proteins called CsrA (carbon storage regulator) or RsmA (regulator of secondary metabolism) are central components of such global regulatory networks that are involved in the transition from exponential to stationary growth phases in several species (1). In *Escherichia coli*, CsrA plays an important role in regulating carbon metabolism and motility (2,3,4) besides also controlling biofilm formation and dispersal (5). CsrA homologs, which have been mostly found in Gram-negative γ-proteobacteria but are also present in some Gram-positive species, are also known to regulate the virulence factors of animal and plant pathogens. This has been documented by a series of studies in several bacterial species such as *Salmonella enterica* serovar Typhimurium*, Pseudomonas aeruginosa, Pseudomonas syringae, Pectobacterium caratovora, Legionella pneumophila* (6,7,8,9,10,) *Borrelia burgdorferi* and *Bacillus subtilis* (11,12). While these studies have explored various cellular functions regulated by CsrA/RsmA homologs, a recent review states that these post-transcriptional regulators play much wider roles in bacteria and regulate cellular functions ‘on a scale that is underappreciated’ (13). The development of tools enabling and expanding discovery of the Csr/Rsm regulon in multiple species can thus significantly advance our knowledge about an important mechanism for global gene regulation in bacteria.

An essential step in unraveling the Csr/Rsm regulon is the elucidation of target genes directly regulated by CsrA homologs. Direct regulation of gene expression by these proteins occurs at the post-transcriptional level when CsrA/RsmA binds to the messenger RNA (mRNA) of target genes (13,14,15,16,17). For repressed targets, CsrA/RsmA binding can lead to inhibition of translation and/or decreased stability of the transcript, whereas activation of targets can occur due to their binding increasing transcript stability by preventing RNase E-mediated cleavage (18). It is noteworthy that the target mRNAs for which CsrA homologs affect translation but not transcript stability will not be detectable by standard transcriptomic assays such as mRNA microarray hybridization or RNA deep sequencing experiments. There is thus a need for approaches enabling the systematic discovery of direct targets of CsrA homologs which will complement the currently used methods.

Recent studies involving small non-coding RNAs that regulate the activity of CsrA/RsmA homologs (by a multiple binding of the protein leading to its titration) have demonstrated that these proteins primarily bind to the sequence motif A(N)GGA in single-stranded mRNA regions (19,20,21,22,23,24). Our previous work demonstrated that computational searches based on locating intergenic regions with high frequencies of the above core binding motif can lead to the identification of experimentally known CsrA/RsmA-regulating non-coding small RNAs (25). Furthermore, this approach also led to predictions for several previously undiscovered CsrA-type regulating small RNAs, and recent results in *L. pneumophila* (26,27,28) have confirmed the predictions made in this species. The success of this approach suggests that a sequence-based strategy can also be useful in identifying target genes directly regulated by CsrA homologs.

We present here a sequence-based approach for identifying direct targets of CsrA/RsmA homologs in bacterial genomes. The approach is based primarily on information from experimental studies of CsrA homologs binding to target mRNAs. For example, studies in *E. coli* have shown how this binding can result in either repression or activation of target gene expression (2,4,29,30,31,32,33). A recent study in *P. aeruginosa* has identified six genes whose expression is directly repressed at the post-trancriptional level due to binding of RsmA to their mRNAs (34). Other bacterial species for which detailed information for CsrA/RsmA binding to target mRNAs is available include *B. subtilis* (12), *Pseudomonas protegens* (35) and *Salmonella* Typhimurium (36). Focusing on genes that are repressed, the targets identified by these studies can be broadly classified into two categories. The first category consists of targets for which there are multiple binding sites for CsrA homologs in a region around the Shine-Dalgarno (SD) sequence. Examples of target genes in this category are *cstA*, *pgaA*, *glgC*, *cel*, *ydeH, sepL, grlR, nhaR, csrA, sdiA* in *E*. *coli* (2,3,4,6,9,11,16,17,30,31,32,33,37), *hcnA* in *P. protegens* (35), PA0081, PA0082, PA0277, PA3732 in *P. aeruginosa* (34) and *hag* in *B. subtilis* (12) and *flaB* in *B. burgdorferi* (38). The second category consists of genes having only a single known binding site around the SD sequence. Examples include *hfq*, *ycdT* in *E*. *coli* (29,31), *stm1987* (*gcpA*), *yhdA* (*csrD*), *stm1697*, *ydiV* in *S.* Typhimurium (36) and PA4492, PA2541 and *pslA* in *P. aeruginosa* (34,39).

The first category of targets is more amenable to identification via computational sequence-based approaches, since searching for targets with only a single binding site for CsrA is likely to yield many false positives due to the similarities between the A(N)GGA motif and the SD sequence. Our approach thus focuses on a sequence-based algorithm for the identification of a ‘subset’ of target genes in the first category that are directly regulated by CsrA homologs, specifically those which can be identified based on the presence of multiple binding sites satisfying certain sequence criteria as detailed below.

Using available experimental information, we propose a search algorithm for the identification of CsrA-regulated targets in a given bacterial genome. This algorithm differs significantly from the one used in our previous study focusing on the identification of small non-coding RNAs regulating CsrA homologs (25), since the identification of potential mRNA targets requires a different sequence-based strategy. Computational implementation of this strategy leads to prediction of several new targets in *E. coli* and *P. aeruginosa*. Four predicted targets in *P. aeruginosa* were tested experimentally and all of these (including the genes coding for PA0122 (RahU), PA1300 and the global regulators AlgU and PqsR) were validated experimentally, indicating that the code is useful in identifying novel targets of CsrA homologs in bacterial genomes. Furthermore, we highlight a subset of our predictions for three other bacterial species in which the role of CsrA/RsmA in cellular regulation has been studied extensively: *S.* Typhimurium*, P. carotovorum* and *L. pneumophila.* The computer program developed in this work (CSRA_TARGET) can thus be used as a tool to generate testable predictions for direct targets of CsrA homologs, thereby opening up several new avenues of research in efforts to analyze global regulation in diverse bacteria.

In the following, experimental data on CsrA binding to mRNA targets which was used in constructing the sequence-based approach for predicting CsrA targets are reviewed.

### Sequence analysis of known targets

The approach used in this study is based on experimental studies showing direct binding of CsrA homologs to target mRNA for the genes detailed in Table 1. Some key experimental observations point toward the distinguishing features of CsrA/RsmA-regulated targets. First, studies have shown that CsrA homologs bind to additional sites that deviate from the consensus A(N)GGA motif [sites with this consensus motif are termed primary; (21)]. These sites have sequence motifs to which CsrA/RsmA can bind to, albeit with lower affinity, e.g. the motif AGAGA (5,17,32). These additional sites are termed secondary in this study, and accordingly an extended list of binding sites for CsrA homologs is provided in Table 2. It is worth noting that the identification of these secondary binding sites is based on experimental evidence, specifically the demonstration of CsrA/RsmA binding to the proposed site for at least one of the mRNA targets listed above. Secondly, it has been found that cooperative effects are critical in CsrA/RsmA binding to target mRNA (30,32). This suggests that the distribution of binding sites on the mRNA, in particular the distance between adjacent binding sites, can play an important role in determining the mRNA targets of CsrA homologs.

**Table 1.** Experimentally validated targets of CsrA homologs for which binding studies to target mRNA have been used in identifying sequence-based constraints used in this study

CsrA repressed targets	Species	References
*pgaA*	*E. coli*	(32)
*cstA*	*E. coli*	(30)
*glgC*	*E. coli*	(2)
*Cel*	*E. coli*	(33)
*ydeH*	*E. coli*	(31)
*hcnA*	*P. protegens*	(35)
*Hag*	*B. subtilis*	(12)

**Table 2.** Primary and secondary binding sites for CsrA homologs considered in this study

Primary binding sites	Secondary binding sites	References
AAGGA	CTGGA	(30)
ACGGA	AGAGA	(2,32)
ATGGA	CGGGA	(35)
AGGGA	TGGGA	(35)
AGGA		

The references provided give evidence for binding to the secondary sites.

Additional insights come from studies analyzing the structure of CsrA/RsmA and its binding to mRNA targets (40,41). A recent study investigating the binding properties of CsrA/RsmA to specifically engineered mRNAs demonstrated that these dimeric proteins can form a bridge complex wherein one protein is bound to two sites within an mRNA (42). The distance between the sites has to be greater than (or equal to) 10 nt, and double binding was demonstrated for sites within an RNA separated by up to 63 nt. The results from this study provide important constraints that guide us in the development of an algorithm for predicting direct targets of CsrA. Specifically, we consider that binding sites on a given mRNA whose separation lies between 10 nt and 60 nt can be bound by a CsrA or an RsmA dimer. Note that the distance between binding sites refers to the ‘linear’ separation at the sequence level; the actual distance may vary depending on mRNA folding and secondary structure. However, an analysis of the predicted secondary structures of the binding regions for known targets reveals no common signatures, thus as a first approximation we ignore secondary structure effects and consider only sequence-based criteria.

Furthermore, for several known targets, there are often instances of adjacent binding sites that are separated by less than 10 nt. Since a CsrA or RsmA dimer is unlikely to bind simultaneously to both of these sites given that the separation is less than the minimum required, a possible functional role for such arrangement could be to act as pairs to effectively increase the likelihood of one of the dimer subunits binding to either of the two sites. Since the secondary sites are expected to bind CsrA with a lower affinity, having an additional binding site nearby (i.e. within 10 nt) is likely to be an important factor controlling potential binding of CsrA/RsmA to that site. Correspondingly, we assume that secondary binding sites should be considered as potential binding sites only if they are located within the distance of 10 nt from another primary or secondary site.

Analyzing the distribution of CsrA binding sites in the known target mRNAs used in this study (Table 1) from the above perspective, the following sequence characteristics are common to all the targets considered: (i) presence of an A(N)GGA binding site in the vicinity of, or overlapping, the SD sequence; (ii) presence of at least three CsrA/RsmA binding sites; (iii) presence of at least two CsrA binding site pairs with distances <60 nt from each other.

The minimal contiguous sequence region containing such a sequence of binding sites is denoted as the ‘binding region’. For a given gene to be a direct target of a CsrA homolog, the binding region must be located downstream of the transcription start site. We propose that additional potential targets of CsrA can be identified by searching for genes with binding regions (located downstream of transcription start sites) satisfying the constraints noted above.

Recent studies on *hcnA* in *P. protegens* (previously *fluorescens*) suggest additional constraints for target regulation by CsrA homologs. While *hcnA* satisfies all the sequence constraints noted above, binding and mutagenesis studies have found that having only the triplet of sites is not sufficient for CsrA homolog binding; additional sites present further upstream (the *hcnA* leader has five such binding sites in all) are required for RsmE-based repression (35). Although RsmE is a second homolog of RsmA present in *P. protegens*, the two proteins are highly similar and their RNA recognition sites appear to be very similar if not identical to those of *E. coli* CsrA due to the high degree of conservation between these homologs (40,41), even if in some cases RsmE has appeared to be a more effective translational repressor than RsmA (35). These additional constraints serve as a guide in the development of a search algorithm for predicting target genes of CsrA homologs.

## MATERIALS AND METHODS

### Outline of search algorithm

**Figure 1.**
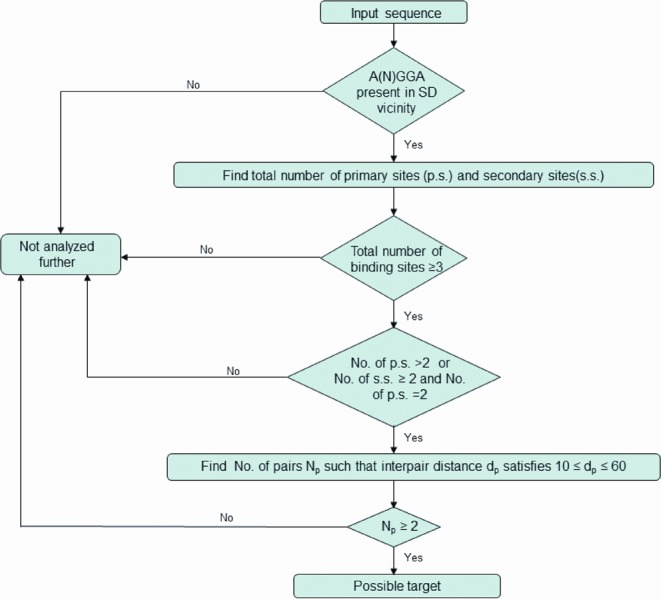
Flowchart for CSRA_TARGET program algorithm.

The observations made on demonstrated CsrA/RsmA target genes motivate the computational search strategy that is outlined in the following. The strategy is designed to identify potential mRNA sequences that have at least two distinct binding configurations for a CsrA homolog dimer. Additional constraints regarding the distribution of primary/secondary sites [see step (iii(b)) below] are derived from observations of the binding of RsmE to the *hcnA* mRNA in *P. protegens*. The flowchart for the proposed algorithm is shown in Figure 1 and further details are the following: for every gene [defined here as an annotated open reading frame (ORF)] in a given bacterial genome sequence, (i) if transcription start sites are known, extract the sequence corresponding to the longest transcript down to 30 nt downstream of the translation initiation codon; or (ii) if transcription start sites are not annotated, consider instead 200 nt upstream and 30 nt downstream of the first codon. With the obtained sequences, identify those that have an A(N)GGA motif in the vicinity of, or overlapping, the SD sequence. Based on analysis done in recent work (43), the SD overlap region is defined as the region from 30 nt upstream of the translation initiation codon to 5 nt into the ORF. For these sequences, find the total number of primary and secondary binding sites (such that the secondary binding sites are all within 10 nt of other sites). Consider all those sequences that have at least three such sites. Then, (iii) among these sequences find the ones that meet one of the following criteria: (a) three or more primary sites or (b) at least two primary sites and two or more secondary sites; (iv) sort out the sequences that have pairs of potential binding sites separated by between 10 and 60 nt. If the number of distinct pairs is greater than or equal to 2, consider it as a potential target.

### Algorithm details and sequence analysis

The computer code (CSRA_TARGET) for identifying CsrA-repressed targets was developed as Perl scripts and is freely available upon request. Intergenic regions and ORFs were obtained from annotated genomic sequences using the Regulatory Sequence Analysis Tools (44). Transcription start sites for *E. coli* genes were obtained from the EcoCyc database (45).

### Construction of *P. aeruginosa* strains in which *rsmA* is constitutively overexpressed or conditionally expressed

To obtain strains PASK09 (*rsmA*^++^) and PASK10 (*rsmA*_IPTG-ind_), two suicide plasmids for allelic replacement were constructed as follows: (i) the *Bam*HI Ω cassette from pHP45Ω (46) was inserted in pSK82 (10) to produce the intermediate plasmid pSK83. The resulting 4.6-kb (P*_rsmA_*-ΩSm^R^/Sp^R^-*lacI*^Q^-P*_tac_*-*rsmA*) *Xho*I–*Xba*I fragment from pSK83 was then subcloned into pDM4 (47) to produce the suicide plasmid pSK11, and (ii) the 1.1-kb (P*_rsmA_*-P*_tac_*-*rsmA*) *Xho*I–*Xba*I fragment from pSK59 (10) was subcloned into pDM4 to generate the suicide plasmid pSK60. Strain PASK09 is a *P. aeruginosa* PAO1 (48) derivative constitutively overexpressing *rsmA*. It was constructed by chromosomal allelic exchange using the suicide plasmid pSK60, resulting in the insertion of the *tac* promoter transcribing the *lacZ* leader and its SD sequence immediately upstream of the *rsmA* ORF, resulting in its strong, constitutive transcription and translation. The construction of conditional *rsmA* mutant strain was similar to that of PASK09 but carried out with the suicide plasmid pSK11: in addition an ΩSm^R^/Spc^R^ interposon to terminate any native transcription originating upstream of the *rsmA* ORF and the *lacI*^Q^ repressor gene were inserted upstream of the P*tac-*SD*_lacZ_*-*rsmA* construct. This resulted in strain PASK10, which exhibits a conditional *rsmA*-negative phenotype that can be switched to wild-type or *rsmA* overexpression levels by supplementing the medium with varying concentrations of isopropyl β-D-1-thiogalactopyranoside (IPTG). Additional details on strains PASK09 and PASK10 are provided in Supplementary Figure S1.

### Bacterial strains and growth conditions

Details of *P. aeruginosa* wild type (PAO1, Nottingham subline), and its derived Δ*rsmA* mutant (PAZH13), *rsmA^++^* over-expresser (PASK09) and IPTG-inducible *rsmA* (PASK10) strains, as well as plasmids used in this study are listed in Table 3. These strains were routinely grown in Luria-Bertani broth (LB) or on tryptic soy agar (TSA) plates. For selection when required, tetracycline (Tc) was added at 10 μg ml^−1^ for *E. coli* and at 100 μg ml^−1^ for *P. aeruginosa*. For qualitative β-galactosidase assays, 50 μg ml^−1^ 5-bromo-4-chloro-3-indolyl-β-D-galactopyranoside (X-gal) and, when required, 1-mM IPTG were added to the medium.

**Table 3.** Bacterial strains, plasmids and oligonucleotides used in this study

Strain, plasmid or oligonucleotide	Genotype/comment	Reference
*P. aeruginosa*		
PAO1	Wild type, University of Nottingham laboratory subline from which the three strains below are derived	()
PAZH13	*rsmA* deletion mutant	()
PASK09	*rsmA* constitutively expressed from a *tac* promoter inserted in the chromosome, obtained by allelic exchange using pSK11 on PAO1	(this study)
PASK10	*rsmA*::ΩSm/Spc-*lacI*^Q^-P*_tac_*-*rsmA*; IPTG-inducible, conditional *rsmA* mutant, obtained by allelic exchange using pSK60 on PAO1	(this study)
*E. coli*		(this study)
Top′10 cells	F- *mcrA* Δ(*mrr-hsdRMS-mcrBC*) Φ80lacZΔM15 Δ*lacχ*74 *recA*1 *araD*139 Δ(araleu) 7697 *galU galK rpsL* (Str^R^) *endA*1 *nupG*	Invitrogen
DH5α	F- *endA*1 *glnV*44 *hsdR*17 *supE*44 *thi*-1 *recA*1 *gyrA*96 *relA*1 *nupG* φ80Δ*lacZ*-M15 Δ(*lacZYA*–*argF*)U169 *deoR*	Invitrogen
**Plasmids**		
pME6014	pVS1-p15A shuttle vector for translational *lacZ* fusions, Tc^R^, Supplementary Figure S2	()
pME6015	pVS1-p15A shuttle vector for translational *lacZ* fusions, Tc^R^, Supplementary Figure S2	()
pME6014_*rahU*	415-bp *Bam*HI and *Pst*I-digested PCR product cloned into *Bam*HI and *Pst*I-digested pME6014. Translational *rahU*’-’*lacZ* fusion at the 16th codon, Tc^R^	(this study)
pME6015_*pqsR*	546-bp *Bam*HI and *Pst*I-digested PCR product cloned into *Bam*HI and *Pst*I-digested pME6015. Translational *pqsR*’-’*lacZ* fusion at the 20th codon, Tc^R^	(this study)
pME6015_*algU*	570-bp *Bam*HI and *Pst*I-digested PCR product cloned into *Bam*HI and *Pst*I-digested pME6015. Translational *algU*’-’*lacZ* fusion at the 20th codon, Tc^R^	(this study)
pME6015_PA1300	562-bp *Bam*HI and *Pst*I-digested PCR product cloned into *Bam*HI and *Pst*I-digested pME6015. Translational PA1300’-’*lacZ* fusion at the 20th codon, Tc^R^	(this study)
pSK11	Suicide plasmid to insert by allelic exchange the P*tac* promoter upstream of *rsmA*, to generate *rsmA*-overexpressing strains, Cm^R^	(this study)
pSK60	Suicide plasmid to insert by allelic exchange a ΩSm^R^/Sp^R^-*lacI*^Q^-P*_tac_* construct upstream of *rsmA*, to generate IPTG-inducible, conditional *rsmA* mutant strains, Cm^R^	(this study)
**Oligonucleotides (5′-3′)**		
*rahU*_target	FP: GCCTGC*GGATCC*CAGCGCGCCCTGCTCGATG, *Bam*HI underlined	(this study)
	RP: CCACCGG*CTGCAG*T**GGA**TTTGGATACCACGACC, *Pst*I underl., 16th codon in bold	
*algU*_target	FP: GCCTGC*GGATCC*ATGCGCAGGTGTTCCGGA, *Bam*HI underlined	(this study)
	RP: CCACCGG*CTGCAG***CCG**CTTGTCTCCGCGCTGTA, *Pst*I underl., 20th codon in bold	
*pqsR*_target	FP: GCCTGC*GGATCC*TAGAACCGTTCCTGGCTCGGC, *Bam*HI underlined	(this study)
	RP: CCACCGG*CTGCAG***CGA**ACCGGAGGCGATGACCTGGAGGAACAT, *Pst*I underlined, 20th codon in bold	
PA1300_target	FP: GCCTGC*GGATCC*AGCTCGAGGACGAGGACGACG, *Bam*HI underlined RP: CCACCGG*CTGCAG***CAA**CTCGCCATGGAACGCCTGATAGGCAT, *Pst*I underlined, 20th codon in bold	(this study)

### Growth curves

A single colony from each plasmid-bearing strain was inoculated in LB medium with Tc and incubated at 37°C at 200 revolutions per minute (rpm) for 18 h, after which they were diluted 1:100 in fresh LB medium with Tc. Growth was then periodically measured at OD_600_. For western blot analysis, *P. aeruginosa* strains were grown for 11 h and samples were collected every hour from 6 h onward, normalizing the bacterial suspensions to an OD_600_ of 0.1 and processing always the same number of bacteria.

### Total proteins from whole-cell lysates

Culture samples of 1 ml were collected at different time points and normalized to an OD_600_ of 0.1 with sterile LB. The cells were then pelleted and resuspended in 75 μl of Laemmli buffer (51), and boiled for 10 min. The cell debris were removed by centrifugation at 20 800 × *g* for 10 min and the resulting clear supernatants constituted the protein extracts.

### Sodium dodecyl sulphate-polyacrylamide gel electrophoresis and Western blot analysis

Equal volumes of 25 μl of protein extracts in Laemmli buffer were separated on 8–16% sodium dodecyl sulphate-polyacrylamide gel electrophoresis gels using the Criterion gel system (Bio-Rad). Proteins were transferred by electroblotting onto 0.2-mm nitrocellulose membranes (Bio-Rad) at 100 V for 45 min. Membranes were blocked with 5% (w/v) fat-free milk in PBS-T [10-mM phosphate buffered saline (PBS) (pH 7.4) with 0.05% Tween-20] for 1 h at room temperature after which blots were probed with anti-recombinant-RahU (PA0122) mouse serum (52) diluted 1:2000 in PBS-T, and incubated overnight at 4°C. Immunodetection was performed with peroxidase-conjugated rabbit anti-mouse immunoglobulin G secondary antibody (Sigma) at a dilution of 1:5000 in PBS-T. The blots were then washed three times with PBS-T followed by PBS for 5 min each. Finally, the peroxidase reaction product was visualized using enhanced chemiluminescence (ECL Kit) according to the manufacturer's protocol (Amersham).

### Construction of *lacZ* translational reporter fusions

Primers for the amplification of selected predicted *rsmA* targets, plasmids and constructs used in this study are listed in Table 3. The *rsmA* target amplicons for *rahU* (415 bp), *algU* (570 bp), *pqsR* (546 bp) and PA1300 (562 bp) each contain the extensive 5′ untranslated region and a putative promoter. The first codons of each target gene (16 for *rahU*, 20 for the three others) were fused in frame with the ‘*lacZ* gene in the reporter vectors pME6014 or pME6015 (50; Supplementary Figure S2). Polymerase chain reaction (PCR)-amplified deoxyribonucleic acid (DNA) fragments corresponding to each target were purified using the Gel Extraction Kit (Qiagen), digested with *Bam*HI and *Pst*I, and inserted into pME6014 or pME6015 plasmids digested with the same enzymes to generate *lacZ* translational reporter fusions for RsmA control analysis. Generated constructs were designated pME6014_*rahU*, pME6015_*algU*, pME6015_*pqsR* and pME6015_PA1300. Inserts obtained by PCR were verified for the absence of unwanted substitutions by sequencing at the Virginia Bioinformatics Institute Core Facility at Virginia Tech. Plasmid constructs were introduced into the *P. aeruginosa* strains PAO1, PAZH13, PASK09 and PASK10 by electroporation and transformants were selected on TSA with Tc plates.

### β-galactosidase assays

Qualitative and quantitative β-galactosidase assays were performed using *P. aeruginosa* strains (PAO1, PAZH13, PASK09 and PASK10) harboring pME6014_*rahU* or pME6015_*algU* (as mentioned in Table 1), as follows: briefly, a single colony from each *P. aeruginosa* strain harboring a translational reporter plasmid was grown in LB medium with Tc for 18 h at 37°C, after which 3 μl were spotted on TSA plates with Tc and X-gal and incubated at 37°C. After 4 h of incubation, 10 μl of sterile water or 1-mM IPTG was added to induce *rsmA* on the PASK10 culture spots on the plate. These plates were then further incubated at 37°C for 48 h and then the blue and white coloration of the spots on the plates was assessed.

Quantitative β-galactosidase assay was performed as follows: all of the *P. aeruginosa* strains (as mentioned above) were grown in LB medium with Tc for 18 h at 37°C and normalized to an optical density (OD_600_) of 0.01 in fresh LB medium and incubated for 11 h with shaking at 37°C. Strain PASK10 was grown either in the absence (uninduced) or in the presence (induced) of IPTG, added at an OD_600_ of 0.5 to a final concentration of 1 mM. The cultures were collected during stationary growth phase (11 h after inoculation), normalized to an OD_600_ of 0.3 and assayed in triplicate. Cell pellets from 1 ml of cultures were resuspended in 100 μl of lysis buffer (100-mM Tris-Hcl [pH 7.8], 30-mM NaH_2_PO_4_, 8-mM dithiothreitol (DTT), 8-mM cyclohexanediaminetetraacetic acid (CDTA), 4% [vol/vol] Triton X-100, 200 μg ml^−1^ of polymyxin B sulfate and 4 mg ml^−1^ of lysozyme) and incubated 45 min at 37°C. The β-galactosidase activities were determined by the method of Miller (53) and calculated by using the formula: Miller units = 1000 × [OD_420_/(*t* ·*v*·OD_600_)], where *t* is the time of reaction in minutes and *v* is the volume of the culture supernatant in milliliter used in the assay (normalized to an OD_600_ of 0.3). All the experimental data in Miller units were expressed as mean and standard deviation (±SD). The same aliquots of individual cell pellets were solubilized in parallel in 100 μl of Laemmli buffer and used in western blotting for the quantification of RahU protein production, as described above.

### Analytical size exclusion chromatography

Analytical size exclusion chromatography (SEC) was used to confirm the dimeric state of the RsmA protein after purification from *E. coli* (54), as well as to monitor binding between RsmA and RNA target sequences. A Superdex 75 HR 10/30 analytical column (GE Life Sciences) was calibrated using a Gel Filtration LMW Calibration Kit (GE Life Sciences), which contained: aprotinin (6.5 kDa), ribonuclease A (13.7 kDa), carbonic anhydrase (29 kDa), ovalbumin (43 kDa), conalbumin (75 kDa) and blue dextran 2000 (2 kDa). Absorbance at 280 nm was monitored to determine the elution volumes of injected samples and apparent molecular weights of species eluted in subsequent analytical SEC experiments. For SEC binding experiments, 50-μM protein and 25-μM RNA samples (Table 4) were used in 50-mM NaCl, 25-mM potassium phosphate buffer set at pH 7.0.

**Table 4.** Ribosome binding sites of the four genes used to validate the predictions in *P. aeruginosa*, aligned with respect to the translation initiation codons

Target RNA	Oligonucleotide (5′-3′)
*rahU* (PA0122)	UUAAC**GGA**GAUCGAC***AUG***
*algU* (PA0762)	GAAGA**GGA**GCUUUC***AUG***
*pqsR* (PA1003)	UAAAA**GGA**AUAAGGG***AUG***
PA1300	GCCGGA**GGA**UGCACGG***AUG***
RsmZ-2 (sRNA)	CCCCGAA**GGA**UCGGGG

The sequences corresponding to the RNA oligonucleotides with GGA motifs used to assess RsmA binding are underlined, as is the sequence of the RsmZ stem-loop 2 (RsmZ-2) which was used as a positive control.

### Isothermal titration calorimetry

Isothermal titration calorimetry (ITC) experiments were recorded on a VP-ITC high sensitivity titration calorimeter (MicroCal, GE Healthcare) at 298 K. RNA and protein samples were degassed at 298 K for 10 min prior to the titration experiments. RNA (125-μM RNA, 50-mM NaCl, 25-mM potassium phosphate buffer pH 7.0) was titrated into a cell containing 1.424 ml of protein solution (5–10-μM protein, 50-mM NaCl, 25-mM potassium phosphate buffer pH 7.0). Titrations consisted of one preliminary injection of 2 μl, followed by 29 injections of 10 μl, with 10-min intervals between injections. A constant stirring speed of 300 rpm ensured rapid mixing during the titration. A reference power of 6 μCal s^−1^ was used. Data were analyzed and fitted to a single-site model using Origin software (MicroCal).

## RESULTS AND DISCUSSION

### Predictions in *E*. *coli*

The algorithm outlined in the previous section was used to predict direct targets of CsrA in *E. coli*. The list of 159 predicted targets is provided in Supplementary Table S1, which also highlights the predictions that are consistent with previous studies analyzing the CsrA regulon in *E. coli* (15,55). Note that there are several predicted targets that have not been reported as direct targets in the previous study analyzing direct binding of CsrA to mRNA targets (15). It would thus be of interest to test a subset of these predictions to see if they are validated as targets under different conditions. A comparison with the predictions and experimental results in (55) suggests that several of such predicted targets from this study could indeed be directly regulated by CsrA.

A flowchart indicating the number of targets meeting the requirements at the different stages of the algorithm is presented in Supplementary Figure S3. Several of the genes predicted to be CsrA targets in *E. coli* are involved in stress response. In particular, genes corresponding to master regulators for a range of stress responses which are characteristically encountered by the bacterium during colonization were identified, e.g. the genes encoding the GadA, GadB and GadE proteins which are involved in the acid stress response (56) and EvgA that regulates acid resistance, osmotic adaptation and drug resistance (57). Furthermore, OsmE is involved in the response to osmotic stress (58) whereas PuuR is involved in putrescine degradation (59) and provides protection against reactive oxygen species that typically cause damage as cells enter stationary phase under aerobic respiration. It is interesting to note that genes encoding proteins involved in anaerobic respiration (HyaA and AdhP) are also predicted to be targets of CsrA. Another intriguing predicted target is the gene for MgsA, a protein that catalyzes the formation of methylglyoxal as a byproduct of glycolysis that is extremely toxic to the cell (60). The production of limited amounts of methylglyoxal plays an important role in controlling the balance of carbon flux in the cell and in reducing the stress associated with the accumulation of sugar phosphates (60). It would be of interest to further examine if CsrA indeed regulates the formation of methylglyoxal by regulating the expression of *mgsA*. The products of other predicted targets are involved in different aspects of metabolism, like SfsB that acts as a transcriptional regulator for maltose metabolism (61).

### Predictions in *P. aeruginosa*

The RsmA (CsrA) pathway regulates secondary metabolism and influences quorum sensing, motility, biofilm formation and virulence in *P. aeruginosa* (62). However the direct targets of RsmA which link to these cellular functions are largely unknown and our results lead to interesting predictions in this context, for example: (i) *algU* encodes an alternative sigma factor that controls alginate production which can lead to mucoidy and chronic infections for cystic fibrosis patients (63); (ii) *pqsR* (also known as *mvfR*) codes for a LysR-type regulator required for the transcription of the *pqsABCDE* and *phnAB* operons and the biosynthesis of 2-alkyl-4(1*H*)-quinolones that play critical roles in quorum sensing and the virulence of *P. aeruginosa* (64); (iii) *rahU* (PA0122) encodes a novel oxidized phospholipid binding protein produced during early stationary phase (52) that potentially plays a role in modulating host innate immunity and biofilm formation (65,66); (iv) PA1300 encodes a σ^70^ factor of the ECF subfamily that was found by transcriptome analysis to be highly induced by iron starvation (67); and (v) *lecA* encodes the galactophilic PA-IL lectin which is a virulence factor that causes damage to respiratory epithelial cells (68). The predicted regulation of *lecA* is consistent with the observation that overexpression of *rsmA* resulted in substantial reduction in the levels of PA-IL lectin (49). Since there are several global regulators among the predicted targets, the results suggest that the number of directly and indirectly regulated targets of RsmA could be quite large. The complete list of 281 predicted targets is provided in Supplementary Table S2, which also highlights the predictions that are consistent with previous transcriptome studies in *P. aeruginosa* (6,34). We note that there are several predicted targets that are not among the list of targets from these previous transcriptome studies. As shown below, some of these targets have now been experimentally validated in this study.

### Experimental validation of novel targets of RsmA in *P. aeruginosa*

We selected a small subset of the predicted targets for experimental validation. One of the targets (*rahU*) has been studied by us in previous work (52,65–66) and hence was a natural target for validation. The remaining targets were chosen either based on their importance as global regulators (*algU, pqsR*) or based on a high concentration of predicted binding sites (PA1300).

**Figure 2.**
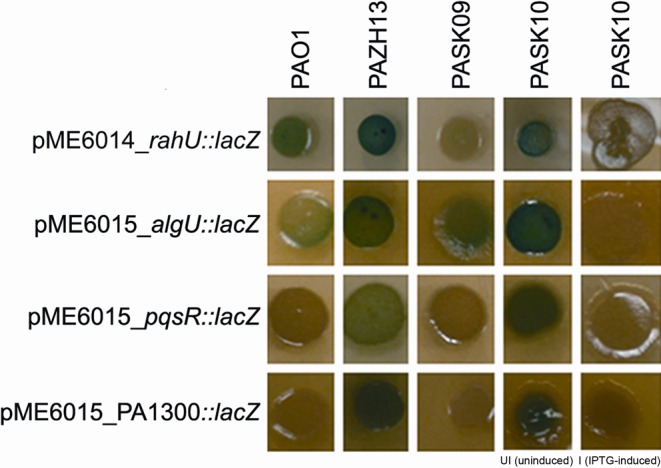
Qualitative β-galactosidase assay for predicted RsmA targets. Regulation of the selected predicted RsmA targets *rahU*, *algU*, *pqsR* and PA1300 in *P. aeruginosa* strains PAO1, PAZH13 (*rsmA* deletion mutant), PASK09 (constitutively overexpressing *rsmA*) and PASK10 (IPTG-inducible, conditional *rsmA* mutant). Translational fusions of these genes with *lacZ* exhibited β-galactosidase activities that varied in the wild-type PAO1 strain (light or no blue coloration) were increased in PAZH13 and uninduced PASK10 (enhanced intensity of the blue color) and were reduced in PAK09 and IPTG-induced PASK10.

The above four predicted targets were cloned and incorporated into translational *‘lacZ* reporter fusions. Each fusion was constructed such that the DNA fragment contained a putative promoter region and the 5′ untranslated transcribed region with the predicted *rsmA* binding sites, as well as the first 16–20 codons (including the ATG start site) of each target gene translated in frame with *‘lacZ*. The β-galactosidase activities of *P. aeruginosa* strains (PAO1, PAZH13, PASK09 and PASK10) harboring the *rsmA* target’-’*lacZ* translational reporter fusion plasmids were qualitatively assessed on TSA plates supplemented with Tc and X-gal (Figure 2). Enhanced β-galactosidase activities were seen for the four fusions in RsmA-deficient strains PAZH13 and PASK10 (uninduced condition) compared to that obtained in the wild-type PAO1 (in which expression levels appeared variable), while in contrast, in RsmA-overproducing strains PASK09 and PASK10 (IPTG-induced) the activities of the reporter fusions were strongly repressed. These results support the prediction that *rahU*, *algU*, *pqsR* and PA1300 are genes that are directly repressed by RsmA at the post-transcriptional level.

### Biophysical analysis of protein–RNA interactions *in vitro*

**Figure 3.**
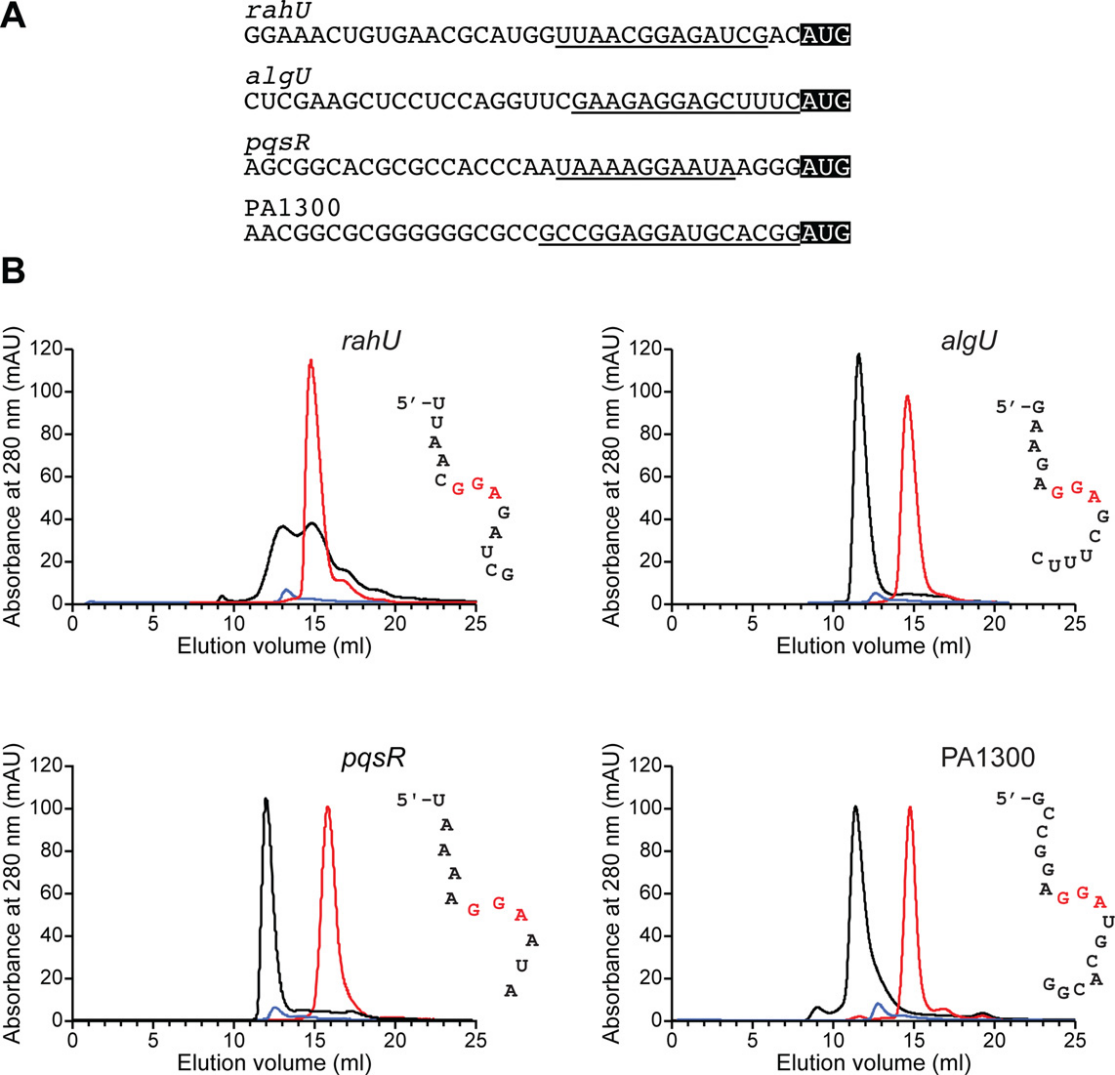
Analytical SEC of RsmA binding to predicted RNA targets. (**A**) Sequences of the ribosome binding regions of *rahU*, *algU*, *pqsR* and PA1300. Start codons are highlighted and the sequences corresponding to the RNA oligonucleotides used in the binding assays are underlined. (**B**) Binding interactions of RsmA determined qualitatively by analytical SEC showing a shift in retention time of the band for unbound RNAs (red) to faster elution for the complexes (black); protein alone shown in blue. The SEC profiles are for the predicted targets of *rahU*, *algU*, *pqsR* and PA1300 underlined in (A) and shown as unstructured oligonucleotides beside each panel with the GGA binding motif highlighted in red. In the case of *rahU*, binding of around 50% of the RNA was achieved in this assay.

To confirm that RsmA was able to repress translation of *rahU*, *algU*, *pqsR* and PA1300 via direct RsmA–mRNA interactions, *in vitro* binding assays were carried out using His-tagged protein RsmA and short synthetic RNA oligonucleotides, the sequences of which were derived from the ribosome binding site regions of the four genes (Figure 3A). The alignment of these sequences with the translation initiation codon (Figure 3A) shows the presence of a GGA motif (as required by the predictive algorithm CSRA_TARGET) within some variation on the ideal SD sequence complementary to the 3′ end of the 16S ribosomal RNA (AGGAGGU). Short RNA molecules (11–17 nt, underlined in Figure 3A) were used rather than more extensive 5′-leader sequences of each gene in order to confirm that these regions were fundamentally sufficient for binding and that it occurred at the ribosome binding site (RBS), removing any uncertainty over the effective sites of interaction with RsmA.

Analytical SEC enables the visualization of complex formation when the binding event causes a sufficiently large increase in size and shape of the RNA to alter its mobility through the gel matrix, with larger molecules eluting before smaller ones. Thus, this technique is well suited to the detection of stable protein–RNA complexes. We first carried out a control experiment with an RNA hairpin, the sequence of which is derived from the regulatory non-coding soluble RNA (sRNA) RsmZ-2 (Supplementary Figure S4). This hairpin carries a 5′-AAGGAU recognition motif within the flexible loop (69) and binds with a *K_d_* = 276 ± 25 nM as measured by ITC analysis (Supplementary Figure S4). An analytical SEC trace, monitoring absorbance at 280 nm of a 50-μM RsmA protein sample with 25-μM RNA, showed the RNA hairpin of RsmZ-2 resulting in a substantial shift in the elution profile when binding to RsmA (Supplementary Figure S4), consistent with an RsmA dimer binding RNA hairpin motifs at each of the two symmetrical sites. Subsequent analysis of an RsmA-R44A mutant, which knocks out a number of key complex stabilizing interactions, virtually eliminated binding as judged by SEC experiments (Supplementary Figure S4) and electrophoretic mobility shift assays (40), without affecting the structural integrity of the RsmA dimer.

We subsequently used this analytical SEC assay to detect complex formation with the four oligonucleotides derived from the ribosome binding regions of *rahU*, *algU*, *pqsR* and PA1300 under the same conditions and concentrations of substrates. The SEC traces for complex formation with the *algU*, *pqsR* and PA1300 RNAs produced single-peak elution profiles corresponding to high affinity complex formation (Figure 3B) consistent with that of the sRNA hairpin of RsmZ-2 (Supplementary Figure S4). Slightly weaker binding by SEC was evident for the *rahU* oligonucleotide in which both the free and bound states were present in a broadened elution profile. In this particular case, this may have resulted from a partial folding or aggregation of the RNA oligonucleotide. Finally, the RsmA-R44A mutant was tested for its ability to bind the same RBS sequences; however, none of the four showed evidence of significant interactions with the mutant with the RNA remaining largely unbound under the same conditions used for the wild-type RsmA protein (data not shown). Thus, we observed specificity in binding the *rahU*, *algU*, *pqsR* and PA1300-derived RNA sequences, which provides further support for RsmA function in sequestering ribosome binding sites in regulating RNA translation.

### The genes *rahU*, *algU, pqsR* and PA1300 are regulated by RsmA in *P. aeruginosa*

**Figure 4.**
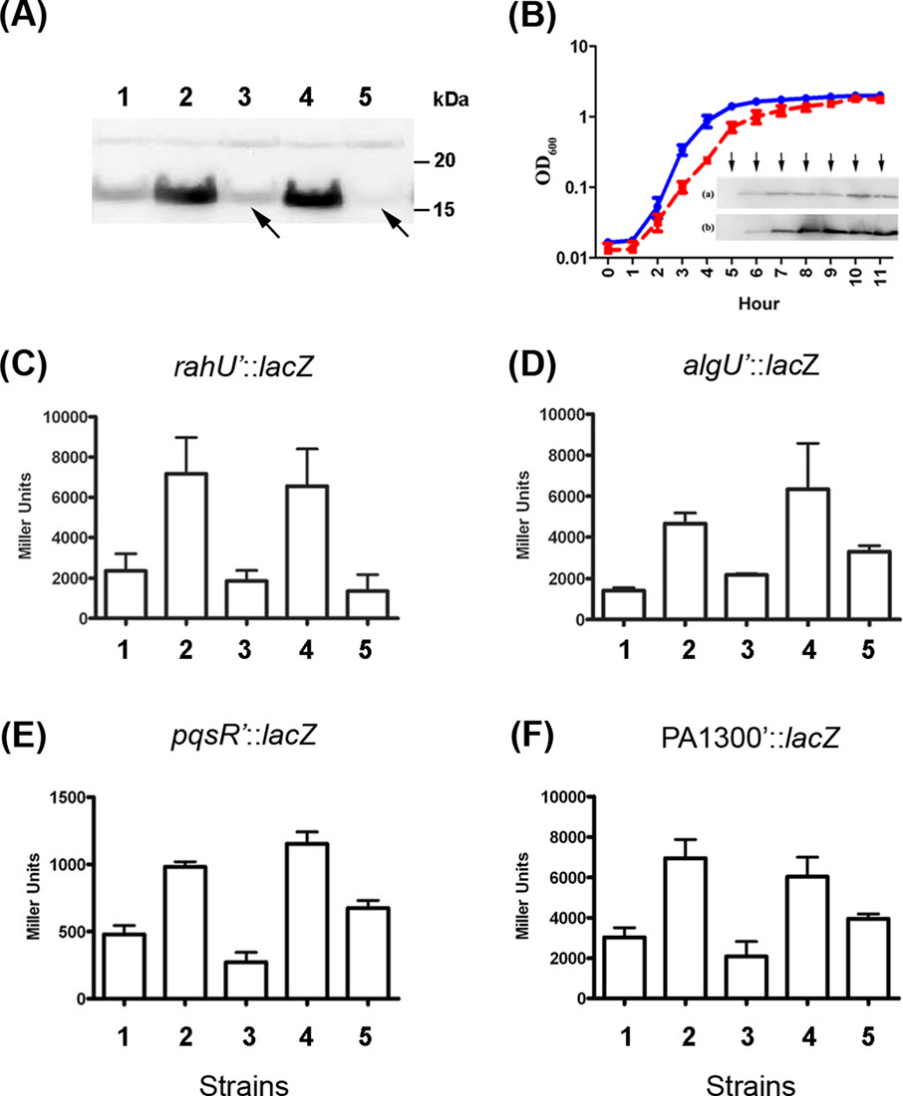
RahU protein production is regulated by RsmA. (**A**) Western blot analysis of RahU production in different constructs: lane 1, PAO1 (wild type); lane 2, PAZH13 (Δ*rsmA*); lane 3 PASK09 (*rsmA*^++^); lane 4, uninduced PASK10 (*rsmA*_IPTG-ind_); and lane 5, PASK10 induced with IPTG. Cells for the assays were collected after 11 h of growth in LB at 37°C with shaking. RahU production was significantly reduced in PASK09 and PASK10-UI strains, when compared to PAZH13 (as shown by arrows). (**B**) RahU production by *P. aeruginosa* strains PAO1 (blue line) and PAZH13 (red line) grown in the same conditions as before. The OD_600_ data shown are from two independent experiments with mean values and ± standard deviation. Total protein extracts from (a) PAO1 and (b) PAZH13 were prepared at regular intervals between 5 and 11 h after inoculation and RahU production was monitored by western blot analysis. The blot results were aligned with the corresponding sampling time points of the growth curves (as marked with down arrows). (**C**)–(**F**) The regulation of the *rahU*’-’*lacZ*, *algU*’-’*lacZ, pqsR’-‘lacZ* and PA1300 translational reporter fusions was confirmed in *P. aeruginosa* strains (as described above, after 11 h of growth). Each bar represents individual strains as in panel (A) and the β-galactosidase activity is plotted in Miller units with mean ± standard deviation from three measurements.

Western blot analysis was carried out on total protein extracted from *P. aeruginosa* strains during stationary growth phase in LB broth (11 h after inoculation, no significant differences in growth yields between the different strains were observed). A 16-kDa immunoreactive band corresponding to RahU was detected with an anti-r-RahU antibody as previously published (52). The amounts of RahU protein produced were observed to be higher in RsmA-deficient strains PAZH13 (49) and PASK10 (uninduced, this study) compared to PAO1 (wild type) during stationary growth phase. On the other hand, very low/undetectable production of RahU was seen in strain PASK09, which constitutively expresses *rsmA* from the *tac* promoter, and in the IPTG-induced strain PASK10 (Figure 4A). These results indicate that RahU is negatively regulated by RsmA in *P. aeruginosa*. Although the *rsmA* mutant strain PAZH13 grew slightly more slowly than the parental PAO1 strain, the enhanced production of RahU in strain PAZH13 compared to PAO1 was observed during stationary phase, 6–11 h after inoculation (Figure 4B). Furthermore, we confirmed by using the translational *rahU*’-’*lacZ* fusion construct in a quantitative β-galactosidase assay that the reporter activity was enhanced 3.0-fold in RsmA-deficient strain PAZH13 when compared to PAO1. This enhanced activity was reduced back 3.9-fold when *rsmA* was constitutively expressed from the *tac* promoter in strain PASK09 (Figure 4C). Similarly, expression of the *rahU*’-’*lacZ* reporter construct was enhanced 4.8-fold in the uninduced strain PASK10 compared to when *rsmA* was induced by the addition of IPTG in the same strain (Figure 4C). These observations on the expression of the translational reporter gene fusions corroborate the western blot results and provide additional support to the prediction that *rahU* is directly regulated by RsmA, which acts as a post-transcriptional repressor of its expression. The translational *algU*’-’*lac*Z fusion construct was also regulated by RsmA, as β-galactosidase activity was enhanced 3.3-fold in RsmA-deficient strain PAZH13 when compared to PAO1, an activity also reduced back 2.1-fold in strain PASK09 expressing *rsmA* from the *tac* promoter. Similarly, expression of the *algU*’-’*lac*Z reporter construct was enhanced by 1.9-fold in the uninduced strain PASK10 compared to when *rsmA* was induced by the addition of IPTG (Figure 4D). The translational *pqsR’-’lac*Z fusion construct behaved similarly with respect to differential levels of *rsmA* expression, as β-galactosidase activity was enhanced 2.1-fold in RsmA-deficient strain PAZH13 when compared to PAO1 and reduced back 3.6-fold when *rsmA* was expressed from the *tac* promoter in strain PASK09. Similarly, expression of the *pqsR’-’lac*Z reporter construct was enhanced by 1.7-fold in the uninduced strain PASK10 compared to when *rsmA* was induced by the addition of IPTG (Figure 4E). The translational PA1300*’-’lac*Z fusion construct was also regulated by RsmA, as β-galactosidase activity was enhanced 2.3-fold in RsmA-deficient strain PAZH13 when compared to PAO1, an activity reduced back 3.3-fold in the P*tac*-*rsmA* strain PASK09. Similarly, expression of the PA1300*’-’lac*Z reporter construct was enhanced by 1.5-fold in the uninduced strain PASK10 compared to when *rsmA* was induced by the addition of IPTG (Figure 4F). Altogether these results indicate that RsmA directly controls the expression of *rahU*, *algU*, *pqsR* and PA1300 at the post-transcriptional level.

### Predictions in other species

The conservation of the CsrA/RsmA binding motif across diverse bacteria suggests that the algorithm presented here can be applied to predict CsrA-regulated genes in a majority of bacteria that have well-conserved CsrA homologs. As more species-specific binding information is obtained, the program can be modified to incorporate alternative parameters. Furthermore, for some bacterial pathogens (e.g. *L. pneumophila*) CsrA is known to play a critical role in controlling virulence factors and in regulating the switch between replicative and transmissive phases (8). However, the molecular and genetic basis for CsrA-based control of virulence is largely unknown in these species and our predictions for targets of CsrA can lead to several interesting hypotheses elucidating virulence. To illustrate this, we have applied the algorithm to predict target genes in three other bacterial pathogens in which the role of CsrA homologs has been studied extensively: *S.* Typhimurium, *L. pneumophila* and *P. carotovorum*. For each case, we selected a subset of predicted targets (five targets for each species) comprising well-characterized genes in the respective species which are discussed further below.

#### S. enterica serovar Typhimurium

CsrA is known to be a critical regulator of invasion genes in *S.* Typhimurium (70). Recent work in this species has further demonstrated global regulation by CsrA which was linked to a coordinated bacterial response to environmental stresses during host colonization (7). Our results are consistent with this scenario and lead to novel testable predictions which can further elucidate how global regulation by CsrA is mediated. For example, one of the predicted targets is hilD, which acts as a master regulator for the induction of invasion genes encoded on the Salmonella pathogenicity island I. A recent review (71) highlights indirect evidence that CsrA binds to the hilD transcript and our results add further support to this prediction by identifying potential CsrA-binding sites in the hilD 5′ untranslated transcribed region. Some other identified targets also play major roles in virulence and metabolism: fimY is a regulator of type I fimbrae implicated in initiating intestinal colonization (72) and also regulates motility and virulence gene expression (73); malF encodes a component of the membrane-associated complex (MalFGK_2_) for maltose transport (74); sipA encodes a type III effector protein that is both necessary and sufficient to induce a proinflammatory response in epithelial cells (75); and uspA encodes a universal stress protein that plays an important role in growth arrest, stress and virulence (76). The complete list of predicted targets is provided in Supplementary Table S3.

#### L. pneumophila

CsrA is a global repressor of *L. pneumophila* transmission phenotypes and an essential activator of intracellular replication (8). Recent work has uncovered the existence of a novel LuxR-type quorum sensing regulator, LqsR, which regulates the expression of genes involved in virulence, motility and cell division (77). Interestingly, lqsR is a predicted target gene using our code. Another important predicted target is fleQ which codes for the master transcriptional regulator of flagellar genes. Previous models suggest regulation of FleQ by CsrA (78) and our results further lend support to this hypothesis by identifying corresponding putative CsrA binding sites. Other potentially interesting targets are sodC that codes for a superoxide dismutase; fimV, which encodes a protein that plays an important role in twitching motility, pigment production and morphology (79) and clpP, which encodes a protease required for optimal growth of *L. pneumophila* at high temperatures and under several other stress conditions: cells devoid of ClpP exhibit cell elongation, incomplete cell division and compromised colony formation (80). The complete list of predicted targets is provided in Supplementary Table S4.

#### P. carotovorum

RsmA functions in this species as a key regulator of extracellular enzyme production, quorum sensing, motility and production of secondary metabolites (81). The predicted targets highlight the links to quorum sensing and plant pathogenesis. Two predicted targets, celV and prtW, are known to be major virulence factors of *P. carotovorum* (82,83,84). Another predicted target, hor, codes for a global regulator that controls carbapenem antibiotic production (85). Recent results provide evidence for regulation of hor by RsmA (86) and our analysis suggests that this regulation is directly mediated. The links to quorum sensing are further highlighted by the predicted regulation of expI which is required for the biosynthesis of quorum sensing signal molecules (87). Additionally, we note that one of the predicted targets is nip, which is also known to be a virulence factor (88). Previous work had suggested that RsmA represses the production of Nip (Necrosis-Inducing Virulence Protein, ECA3087) (89) and our results are consistent with these predictions. It should be noted that the genomic analysis was carried out in *Pectobacterium atrosepticum*; however, the functions for most of the genes discussed above are based on work in *P. carotovorum* subsp. *carotovorum*. The complete list of predicted targets is provided in Supplementary Table S5.

## CONCLUSION

In summary, we have developed a computational algorithm that makes predictions for CsrA/RsmA-repressed genes in bacteria. The central element is the presence of multiple binding sites in the neighborhood of the SD sequence with constraints on the distribution of these binding sites. These constraints are defined based on available experimental data and can be further refined as additional knowledge becomes available.

The analysis proposed focuses on identifying only a ‘subset’ of CsrA/RsmA-regulated targets. Currently known targets of these post-transcriptional regulators can be broadly divided into two categories: (i) those with multiple binding sites within the mRNA and (ii) those with a single binding site or two closely spaced (<10-nt distance) binding sites. Several studies have shown that CsrA homologs form and bind as dimers; hence minimally two binding sites per mRNA are required for optimal CsrA/RsmA-based repression. Recent experiments and structural modeling of the CsrA/RsmA dimer suggest that binding to closely separated sites (<10-nt distance) on a single mRNA is sterically unlikely (41,42). Thus for target genes such as *hfq*, the binding geometry to their mRNAs is likely to be such that each dimer binds two sites on two distinct mRNAs, consistent with the binding stoichiometry demonstrated by recent studies with short mRNA fragments from the *hcnA* leader (35). The focus of this analysis is on identifying a subset of mRNA targets in the first category, such that a CsrA homolog dimer can bind to a single mRNA. We have subsequently validated experimentally with RNA oligonucleotides derived from a number of genes that sequences carrying the GGA recognition motif identified by the algorithm are effectively bound as predicted resulting in stable complex formation in solution. The constraints are further chosen such that there are at least two distinct configurations for binding of a CsrA/RsmA dimer to the mRNA, the rationale being that the likelihood of binding/rebinding is increased due to the presence of multiple options for binding.

The corresponding search strategy leads to several (>100) predicted targets in multiple bacterial species. The targets that were tested in *P. aeruginosa* were all validated with binding and reporter gene expression experiments, indicating that the code can successfully identify new targets in genomes and suggesting that many more targets remain to be discovered. Several of the predicted targets in different species indicate important roles for CsrA homologs in diverse processes ranging from stress response and virulence factor regulation to metabolism. If these predictions are validated in future work, they will pave the way for new insights into the roles of CsrA homologs in regulating lifestyle changes in different bacteria. It would also be of interest to verify the conservation of predicted targets across bacterial species, as it can be expected that advantageous regulations would have a tendency to be maintained during evolution. In future work, we plan to carry out a systematic analysis to further identify promising targets for experimental validation in multiple species. The algorithm will also be modified to expand the subset of identifiable target genes to include the screening of binding sites within ORFs, as CsrA homologs also bind in these mRNA regions of some genes such as *infC* in *P. protegens* (90) or *sdiA* in *E. coli* (37). As more experimental data become available, the current algorithm can be refined and readily generalized accordingly. It is hoped that future work, in combination with experiments and comparative analysis across genomes, will provide a broader perspective on this important pathway for global regulation of gene expression in bacteria.

## SUPPLEMENTARY DATA

Supplementary Data are available at NAR Online.

SUPPLEMENTARY DATA
