# Supplementary material for: A sequence-based approach for prediction of CsrA/RsmA targets in bacteria with experimental validation in Pseudomonas aeruginosa
Source: Nucleic Acids Res. 2014 Apr 29;42(11):6811–25. doi: 10.1093/nar/gku309 (PMC4066749; doi:10.1093/nar/gku309)
Supplement: SUPPLEMENTARY DATA [file supp_42_11_6811__index.html]

A sequence-based approach for prediction of CsrA/RsmA targets in bacteria with experimental validation in Pseudomonas aeruginosa — A sequence-based approach for prediction of CsrA/RsmA targets in bacteria with experimental validation in Pseudomonas aeruginosa — SUPPLEMENTARY DATA 

# A sequence-based approach for prediction of CsrA/RsmA targets in bacteria with experimental validation in *Pseudomonas aeruginosa*

## SUPPLEMENTARY DATA

**Files in this Data Supplement:**

- SUPPLEMENTARY DATA
