# Supplementary material for: A sequence-based approach for prediction of CsrA/RsmA targets in bacteria with experimental validation in Pseudomonas aeruginosa
Source: Nucleic Acids Res. 2014 Apr 29;42(11):6811–25. doi: 10.1093/nar/gku309 (PMC4066749; doi:10.1093/nar/gku309)
Supplement: SUPPLEMENTARY DATA [file supp_gku309_nar-03213-h-2013-File002.docx]

Supplementary Information

**Table S1**: Predicted CsrA targets in *E*. *coli* using the program CSRA_TARGET. Predicted targets which were reported in a previous studies analyzing direct binding of CsrA to mRNA targets (Ref. 15 and Ref. 52) are noted.

| **Name** | **Ref. 15** | **Ref. 52** | **Description** |
| --- | --- | --- | --- |
| *yaaY* |  |  | Predicted protein |
| *rluA* |  |  | Pseudouridine synthase for 23S rRNA (position 746) and tRNAphe(position 32) |
| *leuO* |  |  | Probable transcriptional activator for leuABCD operon |
| *mraW* |  |  | S-adenosyl-dependent methyltransferase activity on membrane-located substrates |
| *ldcC* |  |  | Lysine decarboxylase 2, constitutive |
| *ykfM* |  |  | Hypothetical protein, no homologs |
| *ivy* |  |  | Inhibitor of vertebrate C-lysozyme |
| *yafO* |  | ✓ | Predicted toxin of the YafO-YafN toxin-antitoxin system |
| *yagJ* |  |  | CP4-6 prophage; predicted protein |
| *yagK* |  |  | CP4-6 prophage; conserved protein |
| *ykgG* |  |  | Putative transporter; predicted transporter |
| *yaiT* |  |  | Not classified putative flagellin structural protein interrupted by IS3 |
| *yajI* |  |  | Predicted lipoprotein |
| *panE* |  |  | 2-dehydropantoate reductase, NADPH-specific |
| *cyoB* | ✓ |  | Cytochrome o ubiquinol oxidase subunit I |
| *bolA* |  |  | Regulator of penicillin binding proteins and beta lactamase transcription (morphogene) |
| *ybaL* |  | ✓ | Predicted transporter with NAD(P)-binding Rossmann-fold domain |
| *ybbL* |  | ✓ | Predicted transporter subunit: ATP-binding component of ABC superfamily |
| *tesA* |  |  | Multifunctional acyl-CoA thioesterase I and protease I and lysophospholipase L1 |
| *ybbA* |  |  | Predicted transporter subunit: ATP-binding component of ABC superfamily |
| *sfmA* |  |  | Predicted fimbrial-like adhesin protein |
| *entC* |  |  | Isochorismate synthase |
| *cstA* |  | ✓ | Carbon starvation protein |
| *ahpC* | ✓ |  | Alkyl hydroperoxide reductase, C22 subunit |
| *ybfD* |  | ✓ | Putative DNA ligase; conserved protein |
| *galT* | ✓ | ✓ | Galactose-1-phosphate uridylyltransferase |
| *ybhI* |  |  | Predicted transporter |
| *ybiT* |  |  | Fused predicted transporter subunits of ABC superfamily: ATP-binding components |
| *lolA* |  |  | Chaperone for lipoproteins |
| *mgsA* | ✓ |  | Methylglyoxal synthase |
| *yccX* |  | ✓ | Predicted acylphosphatase |
| *hyaA* | ✓ |  | Hydrogenase 1, small subunit |
| *hyaE* |  |  | Protein involved in processing of HyaA and HyaB proteins |
| *pgaA* |  |  | Predicted outer membrane protein |
| *ycdX* |  |  | Predicted zinc-binding hydrolase |
| *ymdA* |  |  | Predicted protein |
| *ymfA* |  |  | Predicted inner membrane protein |
| *ycgE* |  |  | Predicted DNA-binding transcriptional regulator |
| *ycgK* |  |  | Predicted protein |
| *prmC* |  |  | N5-glutamine methyltransferase, modifies release factors RF-1 and RF-2 |
| *yciQ* | ✓ |  | Predicted inner membrane protein |
| *yciW* |  | ✓ | Putative oxidoreductase; predicted oxidoreductase |
| *puuR* |  | ✓ | DNA-binding transcriptional repressor |
| *ycjM* |  | ✓ | Predicted glucosyltransferase |
| *ompG* |  | ✓ | Outer membrane porin |
| *ydaC* |  |  | Rac prophage; predicted protein |
| *ydbD* |  | ✓ | Predicted protein |
| *gapC* |  |  | GAP dehydrogenase glyceraldehyde-3-phosphate dehydrogenase (second fragment); |
| *cybB* |  |  | Cytochrome b561 |
| *fdnI* | ✓ |  | Formate dehydrogenase-N, nitrate-inducible, cytochrome B556(Fdn) gamma subunit; |
| *yddM* |  |  | Predicted DNA-binding transcriptional regulator |
| *adhP* |  |  | Alcohol dehydrogenase |
| *maeA* |  |  | NAD-linked malate dehydrogenase (malic enzyme); |
| *gadB* |  | ✓ | Glutamate decarboxylase B, PLP-dependent |
| *lsrK* |  |  | Autoinducer-2 (AI-2) kinase |
| *ydeH* |  | ✓ | Conserved protein |
| *cspF* |  | ✓ | Qin prophage; cold shock protein |
| *rem* |  |  | Qin prophage; predicted protein |
| *ynfG* |  | ✓ | Oxidoreductase, Fe-S subunit |
| *dmsD* |  |  | Twin-argninine leader-binding protein for DmsA and TorA |
| *folM* |  | ✓ | Putative oxidoreductase; dihydrofolate reductase isozyme |
| *slyB* |  |  | Outer membrane lipoprotein |
| *osmE* |  |  | DNA-binding transcriptional activator |
| *astE* |  |  | Succinylglutamate desuccinylase |
| *fliK* | ✓ |  | Flagellar hook-length control protein |
| *yeeO* |  |  | Predicted multidrug efflux system |
| *erfK* |  | ✓ | Conserved protein with NAD(P)-binding Rossmann-fold domain |
| *yoeB* |  | ✓ | Toxin of the YoeB-YefM toxin-antitoxin system |
| *thiM* |  |  | Hydoxyethylthiazole kinase |
| *yehT* |  |  | Predicted response regulator in two-component system withYehU |
| *rihB* |  |  | Ribonucleoside hydrolase 2 |
| *ccmF* |  |  | Heme lyase, CcmF subunit |
| *yfaS* |  |  | Hypothetical protein putative membrane protein |
| *yfaW* |  | ✓ | Putative racemase; predicted enolase |
| *yfbH* |  | ✓ | Conserved protein |
| *menC* | ✓ | ✓ | O-succinylbenzoyl-CoA synthase |
| *yfbP* |  |  | Predicted protein |
| *nuoK* |  |  | NADH:ubiquinone oxidoreductase, membrane subunit K |
| *yfcN* |  |  | Conserved protein |
| *yfcU* |  |  | Predicted export usher protein;putative membrane |
| *evgA* |  | ✓ | DNA-binding response regulator in two-component regulatory system with EvgS |
| *yfdY* |  |  | Predicted inner membrane protein |
| *yfeC* |  | ✓ | Predicted DNA-binding transcriptional regulator |
| *ypeB* |  |  | GO_component: cytoplasm [goid 0005737]; predicted protein |
| *yfeS* |  |  | Conserved protein |
| *cysA* |  |  | Sulfate/thiosulfate transporter subunit |
| *ucpA* | ✓ | ✓ | Predicted oxidoredutase, sulfate metabolism protein |
| *yfeX* | ✓ |  | Conserved protein |
| *ypfN* |  |  | Predicted protein |
| *hyfB* |  |  | Hydrogenase 4, membrane subunit |
| *hyfR* | ✓ | ✓ | DNA-binding transcriptional activator, formate sensing |
| *yfgJ* |  |  | Predicted protein |
| *yfgM* |  |  | Conserved protein |
| *yfiO* | ✓ |  | Predicted lipoprotein |
| *csrA* | ✓ | ✓ | Pleiotropic regulatory protein for carbon source metabolism |
| *gutM* |  | ✓ | DNA-binding transcriptional activator of glucitol operon |
| *hycI* |  |  | Protease involved in processing C-terminal end of HycE |
| *ygcL* |  |  | Predicted protein |
| *ygcE* |  |  | Putative kinase; predicted kinase |
| *yqcA* |  | ✓ | Predicted flavoprotein |
| *fucA* |  |  | L-fuculose-1-phosphate aldolase |
| *ygeA* |  |  | Predicted racemase |
| *ygeK* |  |  | Predicted protein putative 2-component transcriptional regulator |
| *ygfM* |  | ✓ | Predicted oxidoreductase |
| *guaD* |  | ✓ | Guanine deaminase |
| *gcvP* |  |  | Glycine decarboxylase, P protein of glycine cleavage system |
| *scpC* |  |  | Propionyl-CoA:succinate-CoA transferase |
| *ygfI* |  |  | Predicted DNA-binding transcriptional regulator |
| *ansB* | ✓ |  | Periplasmic L-asparaginase II |
| *yghA* |  | ✓ | Putative oxidoreductase; |
| *ygjH* |  |  | Conserved protein |
| *yqjB* |  | ✓ | Conserved protein |
| *yqjG* |  |  | Putative transferase |
| *yhaV* |  |  | Conserved protein |
| *yhbT* | ✓ | ✓ | Predicted lipid carrier protein |
| *sfsB* |  | ✓ | DNA-binding transcriptional activator of maltose metabolism |
| *yhcE* |  |  | Predicted protein, N-ter fragment (pseudogene) interrupted by IS5 |
| *yhcG* |  | ✓ | Conserved protein |
| *smg* |  |  | Conserved protein |
| *gspD* | ✓ |  | General secretory pathway component, cryptic |
| *gspM* |  |  | General secretory pathway component, cryptic |
| *bfr* |  |  | Bacterioferritin, iron storage and detoxification protein |
| *kefB* |  |  | Potassium:proton antiporter |
| *hofO* |  |  | Conserved membrane protein |
| *glgC* | ✓ | ✓ | Glucose-1-phosphate adenylyltransferase |
| *yhhA* |  |  | Conserved protein |
| *yrhC* |  |  | Predicted protein fragment (pseudogene) |
| *yhhI* |  | ✓ | Putative receptor |
| *yhiI* | ✓ | ✓ | Predicted HlyD family secretion protein |
| *uspA* | ✓ |  | Universal stress global response regulator |
| *gadE* |  | ✓ | DNA-binding transcriptional activator |
| *gadA* | ✓ | ✓ | Glutamate decarboxylase A, PLP-dependent |
| *yhjG* |  |  | Predicted outer membrane biogenesis protein |
| *tag* |  |  | 3-methyl-adenine DNA glycosylase I, constitutive |
| *yiaD* |  | ✓ | Predicted outer membrane lipoprotein |
| *xylH* |  |  | D-xylose transporter subunit |
| *rfaG* |  |  | Glucosyltransferase I |
| *yidX* |  | ✓ | Predicted lipoproteinC |
| *tnaA* | ✓ |  | Tryptophanase/L-cysteine desulfhydrase, PLP-dependent |
| *yieP* |  |  | Predicted transcriptional regulator |
| *yigE* |  |  | Predicted protein |
| *yihQ* |  | ✓ | Putative glycosidase |
| *fdoI* | ✓ |  | Formate dehydrogenase-O, cytochrome b556 subunit |
| *cytR* | ✓ |  | DNA-binding transcriptional dual regulator |
| *fsaB* |  | ✓ | Fructose-6-phosphate aldolase 2 |
| *zraR* |  |  | Fused DNA-binding response regulator in two-component regulatory system with ZraS: response regulator/sigma54 interaction protein |
| *zur* |  |  | DNA-binding transcriptional repressor, Zn(II)-binding |
| *hflK* |  | ✓ | Modulator for HflB protease specific for phage lambda cII repressor |
| *rnr* |  |  | Exoribonuclease R, RNase R |
| *ytfB* |  |  | Predicted cell envelope opacity-associated protein |
| *yjgN* |  | ✓ | Conserved inner membrane protein |
| *insM* |  |  | Predicted transposase fragment (pseudogene) |
| *fimB* |  | ✓ | Tyrosine recombinase/inversion of on/off regulator of fimA |
| *fimC* |  |  | Chaperone, periplasmic |
| *yjiE* |  |  | Predicted DNA-binding transcriptional regulator |
| *yjiK* |  | ✓ | Conserved protein |
| *yjjM* |  |  | Predicted DNA-binding transcriptional regulator |
| *yjjQ* |  |  | Predicted DNA-binding transcriptional regulator |
| *deoD* |  | ✓ | Purine-nucleoside phosphorylase |

**Table S2**: Predicted RsmA targets in *P*. *aeruginosa* using the program CSRA_TARGET. Predicted targets which were reported in previous transcriptome studies (Ref. 6 and Ref. 34) are noted.

| **Locus tag** | **Gene name** | **Ref. 34** | **Ref. 6** | **Description** |
| --- | --- | --- | --- | --- |
| PA0002 | *dnaN* |  |  | Binds the polymerase to DNA and acts as a sliding clamp |
| PA0021 | *Smf* |  |  | Hypothetical protein |
| PA0035 | *trpA* |  |  | tryptophan synthase subunit alpha |
| PA0042 |  | ✓ |  | Hypothetical protein |
| PA0050 |  | ✓ |  | Hypothetical protein |
| PA0052 |  |  |  | Hypothetical protein |
| PA0054 | *yjiI* | ✓ |  | RNA 2'-phosphotransferase-like protein |
| PA0062 |  |  | ✓ | Hypothetical protein |
| PA0065 |  |  |  | Hypothetical protein |
| PA0070 | *tagQ1* |  |  | Involved in Hcp secretion island I (HSI-I) type VI secretion system |
| PA0080 | *tssJ1* |  |  | Involved in Hcp secretion island I (HSI-I) type VI secretion system |
| PA0090 | *clpV1* | ✓ |  | Protease involved in type VI secretion |
| PA0096 |  |  |  | Hypothetical protein |
| PA0098 |  |  |  | 3-oxoacyl-(acyl carrier protein) synthase |
| PA0122 | *rahU* |  |  | Hypothetical protein |
| PA0135 |  |  |  | Hypothetical protein |
| PA0147 |  |  | ✓ | Putative oxidoreductase |
| PA2967 | *fabG* |  |  | 3-ketoacyl-(acyl-carrier-protein) reductase |
| PA0194 |  |  |  | Hypothetical protein |
| PA0197 | *tonB2* |  |  | Periplasmic protein TonB, links inner and outer membranes |
| PA0229 | *pcaT, kgtP* | ✓ |  | Dicarboxylic acid transporter PcaT |
| PA0251 |  |  |  | Hypothetical protein |
| PA0253 |  |  |  | Putative transcriptional regulator |
| PA0254 |  |  |  | Hypothetical protein |
| PA0260 |  |  |  | Hypothetical protein |
| PA0262 |  |  |  | Hypothetical protein |
| PA0272 |  |  |  | Transcriptional regulator |
| PA0332 |  |  |  | Hypothetical protein |
| PA0457.1 |  |  |  | Hypothetical membrane protein |
| PA0490 |  |  |  | Hypothetical protein |
| PA0511 | *nirJ* |  |  | Heme d1 biosynthesis protein NirJ |
| PA0557 |  |  | ✓ | Hypothetical protein |
| PA0563 |  |  |  | Hypothetical protein |
| PA0602 |  | ✓ |  | Putative binding protein component of ABC transporter |
| PA0637 |  |  |  | Hypothetical protein |
| PA0645 |  |  |  | Hypothetical protein |
| PA0651 | *trpC* |  |  | Indole-3-glycerol-phosphate synthase |
| PA0675 | *vreI* |  |  | ECF subfamily RNA polymerase sigma-70 factor |
| PA0736 |  | ✓ |  | Hypothetical protein |
| PA0762 | *algU, algT* |  |  | RNA polymerase sigma factor AlgU |
| PA0782 | *putA, pruB, pruA* |  |  | Bifunctional proline dehydrogenase/pyrroline-5-carboxylate dehydrogenase |
| PA0786 |  |  | ✓ | Probable transporter |
| PA0805 |  |  |  | Hypothetical protein |
| PA0826 |  |  |  | Hypothetical protein |
| PA0843 | *plcR* |  | ✓ | Phospholipase accessory protein PlcR precursor |
| PA0847 |  |  |  | Hypothetical protein |
| PA0852 | *cbpD* |  |  | Chitin-binding protein CbpD precursor |
| PA0861 |  |  | ✓ | Hypothetical protein |
| PA0896 | *aruF* |  |  | Arginine/ornithine succinyltransferase AI subunit |
| PA0906 |  |  |  | Transcriptional regulator |
| PA0940 |  |  |  | Hypothetical protein |
| PA0994 | *cupC3* |  | ✓ | Usher CupC3 |
| PA0995 | *ogt* |  |  | Methylated-DNA--protein-cysteine methyltransferase |
| PA1003 | *mvfR, pqsR* |  |  | Transcriptional regulator PqsR (also known as MvfR) |
| PA1016 |  |  |  | Thiolase |
| PA1024 |  |  |  | 2-Nitropropane Dioxygenase |
| PA1030 |  |  |  | Hypothetical protein |
| PA1046 |  |  |  | Hypothetical protein |
| PA1056 | *phaD* |  |  | Putative monovalent cation/H+ antiporter subunit D |
| PA1067 |  |  |  | Transcriptional regulator |
| PA1094 | *fliD* |  |  | Flagellar capping protein FliD |
| PA1109 |  |  |  | Transcriptional regulator |
| PA1111 |  |  |  | Hypothetical protein |
| PA1125 | *cobB* |  |  | Cobalamin biosynthetic protein |
| PA1126 |  |  |  | Hypothetical protein |
| PA1151 | *imm2* |  | ✓ | Pyocin S2 immunity protein |
| PA1237 |  |  |  | Multidrug resistance efflux pump |
| PA1300 |  | ✓ |  | ECF subfamily RNA polymerase sigma-70 factor |
| PA1301 |  | ✓ |  | Putative transmembrane sensor |
| PA1318 | *cyoB* |  |  | Cytochrome o ubiquinol oxidase subunit I |
| PA1362 |  |  |  | Hypothetical protein |
| PA1369 |  |  |  | Hypothetical protein |
| PA1371 |  |  |  | Hypothetical protein |
| PA1382 | *xqhB* |  |  | Type II secretion system protein |
| PA1426 |  |  | ✓ | Hypothetical protein |
| PA1433 |  |  |  | Hypothetical protein |
| PA1437 |  |  |  | Putative two-component response regulator |
| PA1461 | *motD* |  |  | Flagellar motor protein MotD |
| PA1494 |  | ✓ |  | Hypothetical protein |
| PA1508 |  |  |  | Hypothetical protein |
| PA1511 |  | ✓ |  | Hypothetical protein |
| PA1519 |  |  |  | Putative transporter |
| PA1525 | *alkB2, alkB* |  | ✓ | Alkane-1-monooxygenase 2 |
| PA1528 | *zipA* | ✓ |  | Cell division protein ZipA |
| PA1531 |  |  |  | Hypothetical protein |
| PA1542 |  |  |  | Hypothetical protein |
| PA1549 | *fixI* |  |  | Cation-transporting P-type ATPase |
| PA1573 | *yijF* |  |  | Hypothetical protein |
| PA1579 |  |  |  | Hypothetical protein |
| PA1645 |  | ✓ |  | Hypothetical protein |
| PA1661 |  |  |  | Hypothetical protein |
| PA1668 |  |  |  | Hypothetical protein |
| PA1680 |  |  |  | Hypothetical protein |
| PA1714 | *exsD* | ✓ |  | ExsD |
| PA1715 | *pscB* |  |  | Type III export apparatus protein |
| PA1751 |  |  |  | Hypothetical protein |
| PA1752 |  |  |  | Probable 2-dehydropantoate 2-reductase |
| PA1753 |  |  |  | Hypothetical protein |
| PA1759 |  |  |  | Transcriptional regulator |
| PA1784 |  |  |  | Hypothetical protein |
| PA1791 |  |  | ✓ | Hypothetical protein |
| PA1806 | *fabI, envM* |  |  | NADH-dependent enoyl-ACP reductase |
| PA1817 |  |  |  | Hypothetical protein |
| PA1837 |  |  |  | Hypothetical protein |
| PA1842 |  |  |  | Hypothetical protein |
| PA1869 |  |  |  | Acyl carrier protein |
| PA1897 |  |  |  | Hypothetical protein |
| PA1921 |  |  |  | Hypothetical protein |
| PA1930 |  |  |  | Putative chemotaxis transducer |
| PA1952 |  |  | ✓ | Hypothetical protein |
| PA1970 |  |  | ✓ | Hypothetical protein |
| PA1973 | *pqqF* |  |  | Pyrroloquinoline quinone biosynthesis protein F |
| PA1983 | *exaB* | ✓ |  | Cytochrome c550 |
| PA2020 | *amrR, mexZ* |  |  | Transcriptional regulator |
| PA2030 |  |  |  | Hypothetical protein |
| PA2036 |  |  |  | Hypothetical protein |
| PA2141 |  |  |  | Hypothetical protein |
| PA2147 | *katE* |  |  | Hydroperoxidase II |
| PA2165 | *glgA* |  | ✓ | Glycogen synthase |
| PA2185 | *katN* |  |  | Non-heme catalase KatN |
| PA2189 |  |  |  | Hypothetical protein |
| PA2208 |  |  |  | Hypothetical protein |
| PA2216 |  |  |  | Hypothetical protein |
| PA2225 |  |  | ✓ | Hypothetical protein |
| PA2230 |  |  |  | Hypothetical protein |
| PA2254 | *pvcA* |  |  | Pyoverdine biosynthesis protein PvcA |
| PA2258 | *ptxR* |  |  | Transcriptional regulator PtxR |
| PA2262 | *kguT* |  |  | Putative 2-ketogluconate transporter |
| PA2275 | *yahK* |  |  | Putative alcohol dehydrogenase (Zn-dependent) |
| PA2277 | *arsR* |  |  | ArsR protein |
| PA2311 |  |  |  | Hypothetical protein |
| PA2316 |  |  |  | Putative transcriptional regulator |
| PA2362 |  |  | ✓ | Hypothetical protein |
| PA2380 |  |  |  | Hypothetical protein |
| PA2384 |  |  |  | Hypothetical protein |
| PA2390 | *pvdT* |  |  | Putative ATP-binding/permease fusion ABC transporter |
| PA2392 | *pvdP* |  |  | PvdP |
| PA2411 |  |  | ✓ | Putative thioesterase |
| PA2419 | *slsA* |  |  | Putative hydrolase |
| PA2420 | *opdJ* |  |  | Porin |
| PA2434 |  |  |  | Hypothetical protein |
| PA2454 |  | ✓ |  | Hypothetical protein |
| PA2464 |  | ✓ | ✓ | Hypothetical protein |
| PA2508 | *catC* |  |  | Uuconolactone delta-isomerase |
| PA2518 | *xylX* |  |  | Toluate 1,2-dioxygenase alpha subunit |
| PA2531 |  |  |  | Putative aminotransferase |
| PA2540 |  | ✓ | ✓ | Hypothetical protein |
| PA2541 |  | ✓ |  | Putative CDP-alcohol phosphatidyltransferase |
| PA2554 |  |  |  | Putative short-chain dehydrogenase |
| PA2560 |  | ✓ |  | Hypothetical protein |
| PA2562 |  |  |  | Hypothetical protein |
| PA2570 | *lecA, pa1L* |  |  | Galactophilic PA-IL lectin LecA |
| PA2581 |  | ✓ |  | Hypothetical protein |
| PA2587 | *pqsH* |  |  | FAD-dependent monooxygenase converting HHQ into PQS |
| PA2657 |  |  |  | Putative two-component response regulator |
| PA2658 |  |  |  | Hypothetical protein |
| PA2723 |  |  |  | Hypothetical protein |
| PA2731 |  |  |  | Hypothetical protein |
| PA2748 | *mapB* | ✓ |  | Methionine aminopeptidase |
| PA2756 |  |  |  | Hypothetical protein |
| PA2780 |  | ✓ |  | Hypothetical protein |
| PA2805 |  |  |  | Hypothetical protein |
| PA2830 | *htpX* | ✓ |  | HtpX, Zn-dependent protease with chaperone function |
| PA2841 |  |  |  | Enoyl-CoA hydratase |
| PA2868 |  |  |  | Hypothetical protein |
| PA2877 |  |  |  | Putative transcriptional regulator |
| PA2889 | *atuD* |  |  | Putative citronellyl-CoA dehydrogenase involved in catabolism of citronellol |
| PA2896 |  |  |  | RNA polymerase sigma factor |
| PA2953 |  | ✓ |  | Electron transfer flavoprotein-ubiquinone oxidoreductase |
| PA2972 | *yceF* |  |  | Maf-like protein |
| PA2982 |  |  |  | Hypothetical protein |
| PA3008 |  |  |  | Hypothetical protein |
| PA3017 |  |  |  | Hypothetical protein |
| PA3055 |  | ✓ |  | Hypothetical protein |
| PA3066 |  |  |  | Hypothetical protein |
| PA3121 | *leuC* |  |  | Isopropylmalate isomerase large subunit |
| PA3141 | *wbpM* |  |  | Nucleotide sugar epimerase/dehydratase WbpM |
| PA3192 | *gltR* |  |  | Two-component response regulator GltR |
| PA3218 |  |  | ✓ | Hypothetical protein |
| PA3221 | *csaA* |  |  | CsaA protein |
| PA3229 |  |  | ✓ | Hypothetical protein |
| PA3240 |  |  |  | Hypothetical protein |
| PA3245 | *minE* |  |  | Cell division topological specificity factor MinE |
| PA3284 |  |  |  | Hypothetical protein |
| PA3317 |  |  |  | Hypothetical protein |
| PA3326 | *clpP2* |  |  | ATP-dependent Clp protease proteolytic subunit |
| PA3327 |  |  |  | Non-ribosomal peptide synthetase |
| PA3334 |  |  |  | Acyl carrier protein |
| PA3335 |  |  |  | Hypothetical protein |
| PA3364 | *amiC* |  |  | Aliphatic amidase expression-regulating protein |
| PA3369 |  |  |  | Hypothetical protein |
| PA3371 |  |  |  | Hypothetical protein |
| PA3398 |  |  |  | Putative transcriptional regulator |
| PA3402 |  |  |  | Hypothetical protein |
| PA3433 | *ywbI* |  |  | Transcriptional regulator |
| PA3439 | *folX* |  |  | D-erythro-7,8-dihydroneopterin triphosphate 2'-epimerase |
| PA3458 |  |  |  | Putative transcriptional regulator |
| PA3459 | *asnB* |  |  | Glutamine amidotransferase |
| PA3481 | *mrp* |  |  | Hypothetical protein |
| PA3484 | *tse3* | ✓ |  | Involved in Hcp secretion island I (HSI-I) type VI secretion system |
| PA3488 |  |  |  | Hypothetical protein |
| PA3508 |  |  |  | Transcriptional regulator |
| PA3516 |  |  |  | Adenylosuccinate lyase |
| PA3520 |  |  |  | Hypothetical protein |
| PA3529 | *tsaA* |  |  | Putative peroxidase |
| PA3536 |  | ✓ |  | Hypothetical protein |
| PA3546 | *algX* |  |  | Alginate biosynthesis protein AlgX |
| PA3565 |  |  | ✓ | Putative transcriptional regulator |
| PA3621 | *fdxA* | ✓ |  | Ferredoxin I |
| PA3630 |  |  |  | Putative transcriptional regulator |
| PA3661 |  | ✓ |  | Hypothetical protein |
| PA3691 |  |  |  | Hypothetical protein |
| PA3693 |  |  |  | Hypothetical protein |
| PA3727 |  |  |  | Hypothetical protein |
| PA3729 |  | ✓ |  | Hypothetical protein |
| PA3731 | *yjfJ* | ✓ |  | Hypothetical protein |
| PA3732 | *yjfI* | ✓ |  | Hypothetical protein |
| PA3739 |  |  |  | Putative sodium/hydrogen antiporter |
| PA3796 |  |  |  | Hypothetical protein |
| PA3848 |  |  |  | Hypothetical protein |
| PA3894 | *opmI* |  |  | Outer membrane protein precursor |
| PA3915 | *moaB1* |  |  | Molybdopterin biosynthetic protein B1 |
| PA3927 |  |  |  | Putative transcriptional regulator |
| PA3930 | *cioA* |  |  | Cyanide insensitive terminal oxidase |
| PA3999 | *dacC, dacD* |  |  | D-ala-D-ala-carboxypeptidase |
| PA4041 |  |  |  | Hypothetical protein |
| PA4056 | *ribD, ribG* |  |  | Riboflavin-specific deaminase/reductase |
| PA4067 | *oprG, yciD, ompW* | ✓ |  | Outer membrane protein OprG precursor |
| PA4086 | *cupB1* |  |  | Fimbrial subunit CupB1 |
| PA4090 |  |  |  | Hypothetical protein |
| PA4112 |  |  |  | Putative sensor/response regulator hybrid |
| PA4182 |  |  |  | Hypothetical protein |
| PA4190 | *pqsL* |  |  | Monooxygenase required for the biosynthesis of 2-alkyl-4-hydroxyquinoline N-oxides |
| PA4197 | *bfiS* |  |  | Histidine kinase regulating C4-dicarboxylate transport system |
| PA4226 | *pchE* | ✓ |  | Dihydroaeruginoic acid synthetase required for pyochelin production |
| PA4236 | *katA, catA* |  |  | Catalase |
| PA4286 |  |  |  | Hypothetical protein |
| PA4311 |  |  |  | Hypothetical protein |
| PA4394 | *yggB* |  |  | Hypothetical protein |
| PA4425 | *yraO* |  |  | Probable phosphoheptose isomerase |
| PA4533 |  |  |  | Hypothetical protein |
| PA4542 | *clpB* |  |  | ClpB protease with chaperone function |
| PA4547 | *pilR* |  |  | Two-component response regulator PilR for type 4 fimbrial biogenesis |
| PA4556 | *pilE* |  |  | Type 4 fimbrial biogenesis protein PilE |
| PA4571 |  | ✓ |  | Putative cytochrome c |
| PA4580 |  |  |  | Hypothetical protein |
| PA4674 | *vapI* |  |  | Hypothetical protein |
| PA4730 | *panC* |  |  | Pantoate-beta-alanine ligase |
| PA4739 |  |  |  | Hypothetical protein |
| PA4773 |  |  |  | Hypothetical protein |
| PA4817 |  |  |  | Hypothetical protein |
| PA4833 |  |  |  | Hypothetical protein |
| PA4842 |  |  |  | Hypothetical protein |
| PA4853 | *fis* | ✓ |  | Transcriptional regulator Fis |
| PA4925 |  |  |  | Hypothetical protein |
| PA4979 |  |  |  | Putative acyl-CoA dehydrogenase |
| PA4998 |  |  |  | Hypothetical protein |
| PA5000 | *wapR* |  |  | Alpha-1,3-rhamnosyltransferase WapR |
| PA5004 |  |  |  | Putative glycosyl transferase |
| PA5016 | *aceF, aceB* |  |  | Dihydrolipoamide acetyltransferase |
| PA5040 | *pilQ* | ✓ |  | Type 4 fimbrial biogenesis outer membrane protein PilQ precursor |
| PA5114 |  | ✓ |  | Hypothetical protein |
| PA5120 |  | ✓ |  | Hypothetical protein |
| PA5149 | *mviM* |  |  | Hypothetical protein |
| PA5191 |  |  |  | Hypothetical protein |
| PA5251 |  |  |  | Hypothetical protein |
| PA5264 |  |  |  | Hypothetical protein |
| PA5266 |  |  |  | Hypothetical protein |
| PA5269 |  |  |  | Hypothetical protein |
| PA5292 | *pchP* |  |  | Phosphorylcholine phosphatase PChP |
| PA5307 |  |  |  | Hypothetical protein |
| PA5318 |  |  |  | Hypothetical protein |
| PA5379 | *sdaB* |  |  | Probable L-serine deaminase/dehydratase |
| PA5392 |  |  | ✓ | Hypothetical protein |
| PA5397 |  |  |  | Hypothetical protein |
| PA5432 |  |  |  | Putative acetyltransferase |
| PA5460 |  |  |  | Hypothetical protein |
| PA5472 |  |  |  | Hypothetical protein |
| PA5514 | *poxB* |  |  | Pyruvate dehydrogenase (cytochrome) |
| PA5547 |  |  |  | Hypothetical protein |
| PA5551 |  |  |  | Hypothetical protein |

**Table S3**: Predicted CsrA targets in *S. typhimurium* using the program CSRA_TARGET.

| **Name** | **Description** |
| --- | --- |
| *ahpC* | alkyl hydroperoxide reductase subunit C |
| *ansP* | L-asparagine transport protein |
| *aroF* | phospho-2-dehydro-3-deoxyheptonate aldolase |
| *asd* | aspartate-semialdehyde dehydrogenase |
| *bolA* | positive transcriptional regulator of morphogenetic pathway |
| *ccdB* | controlled cell death protein; post-segregation toxin; toxin addiction system |
| *ccmA* | ATP-binding protein |
| *clpB* | protein disaggregation chaperone |
| *csdA* | cysteine sulfinate desulfinase |
| *csrA* | carbon storage regulator |
| *cyoB* | cytochrome o ubiquinol oxidase subunit I |
| *cysA* | sulfate/thiosulfate transporter subunit |
| *cysN* | sulfate adenylyltransferase subunit 1 |
| *ddl* | D-alanyl-alanine synthetase A |
| *dsbC* | periplasmic protein with protein disulfide isomerase activity |
| *entC* | synthesizes isochorismate acid from chorismate |
| *exoX* | 3'-5' exonuclease activity on single or double-strand DNA |
| *fdnG* | formate dehydrogenase-N subunit alpha |
| *fdnH* | iron-sulfur beta subunit (AAD13439.1) |
| *fimA* | type-1 fimbrial protein |
| *fimY* | fimbriae Y protein (SW:FIMY_SALTY) |
| *fliK* | flagellar hook-length control protein |
| *fljB* | structural flagella protein |
| *fruF* | phosphoenolpyruvate (PEP)-dependent, sugar transporting phosphotransferase system |
| *galT* | galactose-1-phosphate uridylyltransferase |
| *glgC* | glucose-1-phosphate adenylyltransferase |
| *glpB* | sn-glycerol-3-phosphate dehydrogenase (anaerobic) |
| *glxK* | glycerate kinase |
| *gppA* | guanosine pentaphosphate phosphohydrolase |
| *gshB* | glutathione synthetase |
| *hemK* | N5-glutamine S-adenosyl-L-methionine-dependent methyltransferase |
| *hilD* | invasion protein regulatory protein |
| *hlpA* | periplasmic chaperone |
| *hycI* | involved in the C-terminal processing of the large subunit of hydrogenase 3 HycE |
| *iagB* | invasion protein precursor |
| *ilvH* | acetolactate synthase 3 regulatory subunit |
| *ldcC* | lysine decarboxylase 2 |
| *lpfD* | long polar fimbrial protein |
| *lplA* | lipoate-protein ligase A |
| *malF* | maltose transporter membrane protein |
| *malK* | maltose/maltodextrin transporter ATP-binding protein |
| *malP* | maltodextrin phosphorylase |
| *malS* | periplasmic enzyme that degrades maltodextrins that enter via the outer membrane porin LamB |
| *menC* | O-succinylbenzoate synthase |
| *mig-3* | phage tail assembly protein (gi\|2460256) |
| *mraW* | similar to Escherichia coli apolipoprotein (AAC73193.1) |
| *mraY* | First step of the lipid cycle reactions in the biosynthesis of the cell wall peptidoglycan |
| *nagZ* | beta-hexosaminidase |
| *napB* | citrate reductase cytochrome c-type subunit |
| *narY* | nitrate reductase 2 beta subunit |
| *narZ* | nitrate reductase 2 alpha subunit |
| *nirC* | nitrite transporter NirC |
| *nuoK* | NADH dehydrogenase subunit K |
| *nuoM* | NADH dehydrogenase subunit M |
| *orf5* | putative outer membrane protein |
| *osmE* | osmotically-inducible lipoprotein E |
| *oxyR* | DNA-binding transcriptional regulator OxyR |
| *pabC* | catalyzes the formation of 4-aminobenzoate and pyruvate from 4-amino-4-deoxychorismate |
| *panC* | pantoate--beta-alanine ligase |
| *pduD* | propanediol dehydratase medium subunit |
| *pduJ* | polyhedral body protein |
| *pliC* | lysozyme inhibitor |
| *psd* | phosphatidylserine decarboxylase |
| *PSLT025* | putative cytoplasmic protein |
| *PSLT030* | putative cytoplasmic protein |
| *PSLT106* | stability determinant protein |
| *PSLT107* | putative cytoplasmic protein |
| *pspE* | catalyzes the formation of thiocyanate from thiosulfate and hydrogen cyanide |
| *rcK* | resistance to complement killing |
| *rfbK* | LPS side chain defect; phosphomannomutase |
| *rffH* | glucose-1-phosphate thymidylyltransferase |
| *sbcD* | exonuclease subunit SbcD |
| *sbmC* | DNA gyrase inhibitor |
| *serA* | D-3-phosphoglycerate dehydrogenase |
| *sipA* | secreted effector protein |
| *slyX* | hypothetical protein |
| *smg* | hypothetical protein |
| *spaQ* | needle complex export protein |
| *sptP* | protein tyrosine phosphatase/GTPase activating protein |
| *spvR* | regulator of spv operon |
| *srgC* | putative regulatory protein |
| *ssaN* | type III secretion system ATPase |
| *stdB* | outer membrane usher protein |
| *STM0016* | hypothetical protein |
| *STM0050* | nitrite reductase |
| *STM0056* | oxaloacetate decarboxylase subunit gamma |
| *STM0148* | hypothetical protein |
| *STM0268* | hypothetical protein |
| *STM0382* | permease |
| *STM0409* | hypothetical protein |
| *STM0613* | hydrogenase protein |
| *STM0721* | glycosyl transferase family protein |
| *STM0763.s* | transcriptional regulator |
| *STM0910* | hypothetical protein |
| *STM0920* | Ail/OmpX-like protein |
| *STM0926* | minor tail protein |
| *STM1031* | hypothetical protein |
| *STM1045* | minor tail protein |
| *STM1048* | host specificity protein J |
| *STM1048.1N* | hypothetical protein |
| *STM1082* | regulatory protein |
| *STM1492* | ABC transporter permease |
| *STM1551.1n* | hypothetical protein |
| *STM1583* | hypothetical protein |
| *STM1786* | hydrogenase-1 small subunit |
| *STM1854* | inner membrane protein |
| *STM2133* | hypothetical protein |
| *STM2196* | L-serine dehydratase |
| *STM2275* | regulatory protein |
| *STM2373* | hypothetical protein |
| *STM2400* | inner membrane protein |
| *STM2475* | hypothetical protein |
| *STM2484* | hypothetical protein |
| *STM2552* | hypothetical protein |
| *STM2592* | phage tail component L-like protein |
| *STM2593* | phage tail component M-like protein |
| *STM2595* | minor tail-like protein |
| *STM2605* | head-tail preconnector-like protein |
| *STM2607* | head-to-tail joining-like protein |
| *STM2636* | integrase-like protein |
| *STM2707* | phage tail-like protein |
| *STM2731* | hypothetical protein |
| *STM2750* | PTS system glucitol/sorbitol-specific enzyme II |
| *STM2756* | sugar phosphate aminotransferase |
| *STM2844* | hypothetical protein |
| *STM2902* | hypothetical protein |
| *STM2922* | 3-polyprenyl-4-hydroxybenzoate decarboxylase |
| *STM2943* | hypothetical protein |
| *STM2954.1n* | hypothetical protein |
| *STM2959* | glycerate kinase |
| *STM2986.Sc* | integral membrane protein |
| *STM3038* | metalloendopeptidase |
| *STM3083* | mannitol dehydrogenase |
| *STM3084.S* | regulatory protein |
| *STM3527* | hypothetical protein |
| *STM3558* | death-on-curing protein |
| *STM3599* | anaerobic C4-dicarboxylate transporter |
| *STM3600* | sugar kinase |
| *STM3770* | phosphotransferase system enzyme IIC |
| *STM3863* | permease |
| *STM3940* | inner membrane protein |
| *STM4013.S* | membrane-associated metal-dependent hydrolase |
| *STM4197* | inner membrane protein |
| *STM4214* | hypothetical protein |
| *STM4259* | ABC exporter outer membrane component |
| *STM4302* | hypothetical protein |
| *STM4420* | inner membrane protein |
| *STM4493* | hypothetical protein |
| *STM4518* | inner membrane protein |
| *tag* | 3-methyladenine DNA glycosylase |
| *tesA* | multifunctional acyl-CoA thioesterase I/protease I/lysophospholipase L1 |
| *thiE* | thiamine-phosphate pyrophosphorylase |
| *torR* | DNA-binding transcriptional regulator TorR |
| *traJ* | regulatory protein |
| *ung* | uracil-DNA glycosylase |
| *upp* | uracil phosphoribosyltransferase |
| *ushA* | bifunctional UDP-sugar hydrolase/5'-nucleotidase periplasmic |
| *uspA* | universal stress protein A |
| *vacB* | exoribonuclease R |
| *yaaY* | hypothetical protein |
| *yadI* | PTS enzyme |
| *yaiU* | flagellar protein |
| *yaiV* | DNA-binding transcriptional regulator |
| *yajI* | hypothetical protein |
| *ybaO* | transcriptional regulator |
| *ybbO* | short chain dehydrogenase |
| *ybbS* | DNA-binding transcriptional activator AllS |
| *ybgI* | hydrolase-oxidase |
| *ybhN* | hypothetical protein |
| *ybhP* | hypothetical protein |
| *ybhR* | transporter |
| *ybiR* | transporter |
| *ybjD* | hypothetical protein |
| *ycaR* | hypothetical protein |
| *yccR* | DNA transformation protein |
| *yceH* | hypothetical protein |
| *ycfJ* | hypothetical protein |
| *ycjF* | hypothetical protein |
| *ydgE* | multidrug efflux system protein MdtI |
| *ydiO* | acyl-CoA dehydrogenase |
| *yecH* | hypothetical protein |
| *yedP* | mannosyl-3-phosphoglycerate phosphatase |
| *yfbK* | hypothetical protein |
| *yfcH* | sugar nucleotide epimerase |
| *yfdZ* | aminotransferase |
| *yfeC* | negative regulator |
| *yfgM* | inner membrane protein |
| *yfiO* | outer membrane protein assembly complex subunit YfiO |
| *ygdI* | lipoprotein |
| *ygiW* | outer membrane protein |
| *yhcB* | cytochrome d ubiquinol oxidase subunit III |
| *yhhY* | acetyltransferase YhhY |
| *yhiI* | hypothetical protein |
| *yiaB* | inner membrane protein |
| *yicJ* | transporter |
| *yigA* | hypothetical protein |
| *yigN* | DNA recombination protein RmuC |
| *yihP* | GPH family transport protein |
| *yjbQ* | hypothetical protein |
| *yjdL* | di-/tripeptide transport protein |
| *yjiE* | DNA-binding transcriptional regulator |
| *yjjP* | hypothetical protein |
| *yjjQ* | transcriptional regulator |
| *yliA* | glutathione transporter ATP-binding protein |
| *yncC* | DNA-binding transcriptional regulator |
| *yqiA* | Displays esterase activity toward palmitoyl-CoA and pNP-butyrate |
| *yqjG* | glutathione S-transferase |
| *zraP* | zinc resistance protein |

**Table S4**: Predicted CsrA targets in *L. pneumophila* using the program CSRA_TARGET.

| **Name** | **Description** |
| --- | --- |
| *aroE* | shikimate 5-dehydrogenase |
| *astD* | succinylglutamic semialdehyde dehydrogenase |
| *ccmB* | heme exporter protein CcmB |
| *clpP* | ATP-dependent Clp protease proteolytic subunit |
| *csrA* | carbon storage regulator |
| *dcoA* | pyruvate carboxylase subunit B |
| *dnaN* | DNA polymerase III, beta chain |
| *fimV* | FimV protein |
| *fleN* | hypothetical protein |
| *fleQ* | transcriptional regulator FleQ |
| *fliE* | flagellar hook-basal body complex protein |
| *fliQ* | flagellar biosynthetic protein FliQ |
| *folB* | hypothetical protein |
| *gcdH* | hypothetical protein |
| *gcsB* | glycine dehydrogenase subunit 2 |
| *gcvH* | glycine cleavage system protein H |
| *hflC* | membrane protease subunit HflC |
| *hisF* | hypothetical protein |
| *hutU* | urocanate hydratase |
| *hypD* | hydrogenase expression/formation protein HypD |
| *hypE* | hydrogenase expression/formation protein HypE |
| *IscS* | hypothetical protein |
| *katB* | catalase-peroxidase KatB |
| *lpp0012* | hypothetical protein |
| *lpp0050* | hypothetical protein |
| *lpp0067* | hypothetical protein |
| *lpp0082* | hypothetical protein |
| *lpp0103* | hypothetical protein |
| *lpp0104* | hypothetical protein |
| *lpp0142* | hypothetical protein |
| *lpp0144* | hypothetical protein |
| *lpp0151* | hypothetical protein |
| *lpp0190* | hypothetical protein |
| *lpp0220* | hypothetical protein |
| *lpp0228* | hypothetical protein |
| *lpp0255* | hypothetical protein |
| *lpp0266* | hypothetical protein |
| *lpp0270* | hypothetical protein |
| *lpp0317* | hypothetical protein |
| *lpp0318* | hypothetical protein |
| *lpp0359* | formate dehydrogenase |
| *lpp0368* | hypothetical protein |
| *lpp0442* | hypothetical protein |
| *lpp0452* | hypothetical protein |
| *lpp0488* | hypothetical protein |
| *lpp0490* | hypothetical protein |
| *lpp0585* | hypothetical protein |
| *lpp0606* | hypothetical protein |
| *lpp0648* | hypothetical protein |
| *lpp0649* | hypothetical protein |
| *lpp0678* | hypothetical protein |
| *lpp0680* | hypothetical protein |
| *lpp0685* | hypothetical protein |
| *lpp0706* | hypothetical protein |
| *lpp0725* | hypothetical protein |
| *lpp0726* | hypothetical protein |
| *lpp0728* | acetoacetate decarboxylase |
| *lpp0737* | hypothetical protein |
| *lpp0799* | hypothetical protein |
| *lpp0809* | hypothetical protein |
| *lpp0821* | hypothetical protein |
| *lpp0836* | hypothetical protein |
| *lpp0884* | hypothetical protein |
| *lpp0890* | hypothetical protein |
| *lpp0893* | hypothetical protein |
| *lpp0942* | hypothetical protein |
| *lpp0955* | hypothetical protein |
| *lpp0962* | hypothetical protein |
| *lpp0982* | hypothetical protein |
| *lpp0996* | hypothetical protein |
| *lpp1012* | hypothetical protein |
| *lpp1033* | hypothetical protein |
| *lpp1046* | hypothetical protein |
| *lpp1047* | hypothetical protein |
| *lpp1051* | hypothetical protein |
| *lpp1057* | hypothetical protein |
| *lpp1095* | hypothetical protein |
| *lpp1138* | hypothetical protein |
| *lpp1145* | hypothetical protein |
| *lpp1150* | hypothetical protein |
| *lpp1158* | hypothetical protein |
| *lpp1163* | hypothetical protein |
| *lpp1166* | acetylornithine deacetylase |
| *lpp1168* | hypothetical protein |
| *lpp1212* | hypothetical protein |
| *lpp1218* | hypothetical protein |
| *lpp1219* | hypothetical protein |
| *lpp1221* | hypothetical protein |
| *lpp1236* | hypothetical protein |
| *lpp1238* | hypothetical protein |
| *lpp1304* | hypothetical protein |
| *lpp1336* | hypothetical protein |
| *lpp1340* | hypothetical protein |
| *lpp1377* | hypothetical protein |
| *lpp1382* | hypothetical protein |
| *lpp1490* | hypothetical protein |
| *lpp1521* | hypothetical protein |
| *lpp1538* | hypothetical protein |
| *lpp1554* | hypothetical protein |
| *lpp1563* | hypothetical protein |
| *lpp1578* | hypothetical protein |
| *lpp1607* | hypothetical protein |
| *lpp1617* | hypothetical protein |
| *lpp1619* | hypothetical protein |
| *lpp1625* | hypothetical protein |
| *lpp1638* | hypothetical protein |
| *lpp1680* | hypothetical protein |
| *lpp1715* | hypothetical protein |
| *lpp1718* | hypothetical protein |
| *lpp1729* | hypothetical protein |
| *lpp1757* | hypothetical protein |
| *lpp1786* | hypothetical protein |
| *lpp1790* | hypothetical protein |
| *lpp1791* | hypothetical protein |
| *lpp1808* | D-tyrosyl-tRNA(Tyr) deacylase |
| *lpp1818* | hypothetical protein |
| *lpp1822* | hypothetical protein |
| *lpp1835* | hypothetical protein |
| *lpp1844* | hypothetical protein |
| *lpp1848* | hypothetical protein |
| *lpp1897* | hypothetical protein |
| *lpp1900* | hypothetical protein |
| *lpp1915* | hypothetical protein |
| *lpp1916* | hypothetical protein |
| *lpp1917* | hypothetical protein |
| *lpp1918* | hypothetical protein |
| *lpp1962* | hypothetical protein |
| *lpp1973* | hypothetical protein |
| *lpp2001* | hypothetical protein |
| *lpp2023* | hypothetical protein |
| *lpp2043* | hypothetical protein |
| *lpp2063* | hypothetical protein |
| *lpp2085* | hypothetical protein |
| *lpp2115* | hypothetical protein |
| *lpp2137* | hypothetical protein |
| *lpp2146* | hypothetical protein |
| *lpp2163* | hypothetical protein |
| *lpp2237* | hypothetical protein |
| *lpp2246* | hypothetical protein |
| *lpp2254* | hypothetical protein |
| *lpp2261* | hypothetical protein |
| *lpp2271* | hypothetical protein |
| *lpp2272* | hypothetical protein |
| *lpp2327* | hypothetical protein |
| *lpp2329* | hypothetical protein |
| *lpp2336* | hypothetical protein |
| *lpp2337* | hypothetical protein |
| *lpp2341* | hypothetical protein |
| *lpp2342* | hypothetical protein |
| *lpp2346* | hypothetical protein |
| *lpp2396* | hypothetical protein |
| *lpp2407* | hypothetical protein |
| *lpp2433* | hypothetical protein |
| *lpp2441* | hypothetical protein |
| *lpp2449* | hypothetical protein |
| *lpp2478* | hypothetical protein |
| *lpp2494* | malonate decarboxylase subunit beta |
| *lpp2527* | hypothetical protein |
| *lpp2529* | hypothetical protein |
| *lpp2535* | hypothetical protein |
| *lpp2558* | hypothetical protein |
| *lpp2594* | hypothetical protein |
| *lpp2621* | hypothetical protein |
| *lpp2622* | hypothetical protein |
| *lpp2719* | hypothetical protein |
| *lpp2733* | hypothetical protein |
| *lpp2734* | hypothetical protein |
| *lpp2750* | hypothetical protein |
| *lpp2788* | hypothetical protein |
| *lpp2807* | hypothetical protein |
| *lpp2866* | hypothetical protein |
| *lpp2872* | hypothetical protein |
| *lpp2874* | hypothetical protein |
| *lpp2884* | hypothetical protein |
| *lpp2901* | hypothetical protein |
| *lpp2905* | hypothetical protein |
| *lpp2917* | hypothetical protein |
| *lpp2918* | hypothetical protein |
| *lpp2921* | pteridine reductase |
| *lpp2929* | hypothetical protein |
| *lpp2940* | hypothetical protein |
| *lpp2952* | hypothetical protein |
| *lpp2985* | hypothetical protein |
| *lpp2999* | hypothetical protein |
| *lpp3000* | hypothetical protein |
| *lpp3008* | hypothetical protein |
| *lpp3011* | hypothetical protein |
| *lpp3024* | hypothetical protein |
| *lpp3028* | hypothetical protein |
| *lpp3047* | hypothetical protein |
| *lpp3064* | hypothetical protein |
| *lpxA* | UDP-N-acetylglucosamine acyltransferase |
| *lpxK* | tetraacyldisaccharide 4'-kinase |
| *lrsB* | arsenite efflux membrane component-like protein |
| *lspH* | type II secretory pathway protein LspH |
| *lssY* | secretion system protein Y |
| *lvhB11* | hypothetical protein |
| *lvrA* | hypothetical protein |
| *metK* | S-adenosylmethionine synthetase |
| *minE* | cell division topological specificity factor MinE |
| *mraW* | S-adenosyl-methyltransferase MraW |
| *mraY* | phospho-N-acetylmuramoyl-pentapeptide- transferase |
| *mreD* | rod shape-determining protein MreD |
| *parB* | hypothetical protein |
| *pheT* | phenylalanyl-tRNA synthetase subunit beta |
| *pilC* | pilus assembly protein PilC [YP_123804.1;pilC;pilC;lpp1480;3117780;54297435;YP_123804.1;YP_123804] |
| *pilM* | Tfp pilus assembly protein |
| *pilZ* | hypothetical protein |
| *plpp0020* | hypothetical protein |
| *plpp0041* | hypothetical protein |
| *plpp0043* | hypothetical protein |
| *plpp0056* | hypothetical protein |
| *plpp0100* | hypothetical protein |
| *plpp0114* | hypothetical protein |
| *pmbA* | hypothetical protein |
| *pnaB* | NAD(P) transhydrogenase subunit beta |
| *pntA* | pyridine nucleotide transhydrogenase, alpha subunit |
| *ppt* | hypoxanthine-guanine phosphoribosyltransferase |
| *prpB* | 2-methylisocitrate lyase |
| *pyrE* | orotate phosphoribosyltransferase |
| *qxtB* | hypothetical protein |
| *ruvA* | Holliday junction DNA helicase RuvA |
| *sahH* | S-adenosyl-L-homocysteine hydrolase |
| *sodC* | superoxide dismutase [Cu-Zn] precursor |
| *stuC* | sensor histidine kinase |
| *thrS* | threonyl-tRNA synthetase |
| *umuD* | hypothetical protein |

**Table S5**: Predicted CsrA targets in *P. carotovorum* using the program CSRA_TARGET.

| **Name** | **Description** |
| --- | --- |
| *afuA* | ABC transporter iron-binding protein |
| *arbB* | 6-phospho-beta-glucosidase |
| *arnT* | 4-amino-4-deoxy-L-arabinose transferase |
| *budC* | acetoin reductase |
| *ccmH2* | cytochrome C-type biogenesis protein |
| *celV* | endoglucanase V |
| *col* | colicin |
| *cutF* | lipoprotein involved with copper homeostasis and adhesion |
| *cysN* | sulfate adenylyltransferase subunit 1 |
| *dedD* | hypothetical protein |
| *deoR* | DNA-binding transcriptional repressor DeoR |
| *dgt* | deoxyguanosinetriphosphate triphosphohydrolase |
| *ECA0046* | putative signaling membrane protein |
| *ECA0123* | putative lipoprotein |
| *ECA0280* | succinate-semialdehyde dehydrogenase [NADP+] |
| *ECA0352* | AraC family transcription regulator |
| *ECA0371* | putative transcriptional regulator |
| *ECA0378* | hypothetical protein |
| *ECA0428* | putative porin |
| *ECA0434* | methyl-accepting chemotaxis protein |
| *ECA0470* | hypothetical protein |
| *ECA0484* | hypothetical protein |
| *ECA0509* | hypothetical protein |
| *ECA0520* | hypothetical protein |
| *ECA0563* | hypothetical protein |
| *ECA0582* | putative plasmid-related protein |
| *ECA0587* | hypothetical protein |
| *ECA0594* | hypothetical protein |
| *ECA0597* | hypothetical protein |
| *ECA0637* | hypothetical protein |
| *ECA0870* | putative transcriptional regulator |
| *ECA0926* | putative biopolymer transport protein |
| *ECA0931* | putative avirulence protein |
| *ECA1062* | hypothetical protein |
| *ECA1069* | hypothetical protein |
| *ECA1072* | glutathione S-transferase |
| *ECA1153* | hypothetical protein |
| *ECA1172* | hypothetical protein |
| *ECA1351* | putative allophanate hydrolase subunit 1 |
| *ECA1376* | hypothetical protein |
| *ECA1445* | hypothetical protein |
| *ECA1457* | putative thiamine pyrophosphate-dependent protein |
| *ECA1521* | hypothetical protein |
| *ECA1590* | hypothetical protein |
| *ECA1596* | hypothetical protein |
| *ECA1652* | transposase |
| *ECA1849* | GntR family transcriptional regulator |
| *ECA1891* | hypothetical protein |
| *ECA1914* | ABC transporter, ATP-binding protein |
| *ECA1916* | hypothetical protein |
| *ECA1994* | hypothetical protein |
| *ECA2004* | utative lipoprotein |
| *ECA2104* | VgrG protein |
| *ECA2108* | putative lipoprotein |
| *ECA2109* | putative lipoprotein |
| *ECA2110* | hypothetical protein |
| *ECA2125* | hypothetical protein |
| *ECA2212* | putative cyclopropane-fatty-acyl-phospholipid synthase |
| *ECA2241* | hypothetical protein |
| *ECA2388* | hypothetical protein |
| *ECA2457* | hypothetical protein |
| *ECA2523* | hypothetical protein |
| *ECA2558* | hypothetical protein |
| *ECA2641* | permease |
| *ECA2660* | hypothetical protein |
| *ECA2715* | putative esterase |
| *ECA2754* | putative prophage primase |
| *ECA2950* | TetR family transcriptional regulator |
| *ECA3012* | acetyltransferase |
| *ECA3080* | hypothetical protein |
| *ECA3087* | hypothetical protein |
| *ECA3166* | hypothetical protein |
| *ECA3218* | hypothetical protein |
| *ECA3222* | hypothetical protein |
| *ECA3231* | hypothetical protein |
| *ECA3244* | hypothetical protein |
| *ECA3245* | methyl-accepting chemotaxis protein |
| *ECA3255* | two-component system response regulator |
| *ECA3346* | hypothetical protein |
| *ECA3407* | DnaG primase-like protein |
| *ECA3416* | DnaG primase-like protein |
| *ECA3421* | Rhs protein |
| *ECA3426* | putative phospholipase |
| *ECA3427* | hypothetical protein |
| *ECA3440* | hypothetical protein |
| *ECA3479* | hypothetical protein |
| *ECA3499* | amidase |
| *ECA3549* | putative signaling protein |
| *ECA3604* | hypothetical protein |
| *ECA3629* | hypothetical protein |
| *ECA3709* | putative phage-related membrane protein |
| *ECA3710* | putative phage-related lipoprotein |
| *ECA3747* | ABC transporter, membrane spanning protein |
| *ECA3769* | putative pyruvate formate-lyase activating enzyme |
| *ECA3841* | transcriptional regulator SgrR |
| *ECA3843* | LysR family transcriptional regulator |
| *ECA3868* | hypothetical protein |
| *ECA3872* | dihydrodipicolinate reductase |
| *ECA3984* | putative sodium:sulfate symporter |
| *ECA4055* | ypothetical protein |
| *ECA4114* | putative lysine/ornithine decarboxylase |
| *ECA4127* | putative permease |
| *ECA4142* | putative RHS accessory genetic element |
| *ECA4182* | hypothetical protein |
| *ECA4357* | putative lipoprotein |
| *ECA4397* | hypothetical protein |
| *ECA4429* | putative transposase |
| *ECA4458* | putative lipoprotein |
| *ECA4459* | putative lipoprotein |
| *ECA4501* | putative AMP-binding enzyme |
| *expI* | N-acylhomoserine lactone synthesis protein |
| *fliK* | flagellar hook-length control protein |
| *fliZ* | flagella biosynthesis protein FliZ |
| *flxA* | hypothetical protein |
| *glpB* | anaerobic glycerol-3-phosphate dehydrogenase subunit B |
| *gltL* | glutamate/aspartate transport ATP-binding protein |
| *greA* | transcription elongation factor GreA |
| *hasA* | extracellular heme-binding protein |
| *hemK* | N5-glutamine S-adenosyl-L-methionine-dependent methyltransferase |
| *hemN* | coproporphyrinogen III oxidase |
| *hflC* | FtsH protease regulator HflC |
| *hflK* | FtsH protease regulator HflK |
| *hor* | transcriptional regulator SlyA |
| *hrcC* | type III secretion protein |
| *hrpS* | sigma-54-dependent enhancer-binding protein |
| *hyfD* | hydrogenase 4 subunit D |
| *ibpA* | heat shock protein IbpA |
| *ilvH* | acetolactate synthase 3 regulatory subunit |
| *lgt* | prolipoprotein diacylglyceryl transferase |
| *lhr* | putative ATP-dependent helicase Lhr |
| *ligB* | NAD-dependent DNA ligase LigB |
| *luxS* | S-ribosylhomocysteinase |
| *manY* | mannose-specific IIC component |
| *menC* | O-succinylbenzoate synthase |
| *mraW* | S-adenosyl-methyltransferase MraW |
| *mukB* | cell division protein MukB |
| *nfxB* | transcriptional regulator of multidrug resistance genes |
| *nifQ* | nitrogen fixation protein |
| *ntpA* | dATP pyrophosphohydrolase |
| *ogt* | methylated-DNA--protein-cysteine methyltransferase |
| *oppA1* | periplasmic oligopeptide-binding protein |
| *outF* | general secretion pathway protein F |
| *outH* | general secretion pathway protein H |
| *outL* | general secretion pathway protein L |
| *pepA* | leucyl aminopeptidase |
| *pepP* | proline aminopeptidase P II |
| *pepQ* | proline dipeptidase |
| *phnK* | phosphonate C-P lyase system protein PhnK |
| *potF* | putrescine transporter subunit: periplasmic-binding component of ABC superfamily |
| *ppc* | phosphoenolpyruvate carboxylase |
| *ppdD* | putative major pilin subunit |
| *ppiC* | peptidyl-prolyl cis-trans isomerase C |
| *proW* | glycine betaine transporter membrane protein |
| *prtF* | protease secretion protein |
| *prtW* | metalloprotease |
| *purK* | phosphoribosylaminoimidazole carboxylase ATPase subunit |
| *rffC* | TDP-fucosamine acetyltransferase |
| *ribD* | uracil reductase |
| *rimK* | ribosomal protein S6 modification protein |
| *rpoB* | DNA-directed RNA polymerase subunit beta |
| *rseB* | periplasmic negative regulator of sigmaE |
| *sfsB* | sugar fermentation stimulation protein |
| *sirB2* | hypothetical protein |
| *slt* | lytic murein transglycosylase |
| *smg* | hypothetical protein |
| *sppA* | protease 4 |
| *thiE* | thiamine-phosphate pyrophosphorylase |
| *togB* | periplasmic pectic oligomers binding protein |
| *traF* | putative plasmid transfer protein |
| *trpD* | anthranilate phosphoribosyltransferase |
| *truB* | tRNA pseudouridine synthase B |
| *uppS* | undecaprenyl pyrophosphate synthetase |
| *uspA* | universal stress protein A |
| *wza* | putative polysaccharide export protein |
| *xylH* | xylose transport system permease |
| *zitB* | zinc transporter ZitB |


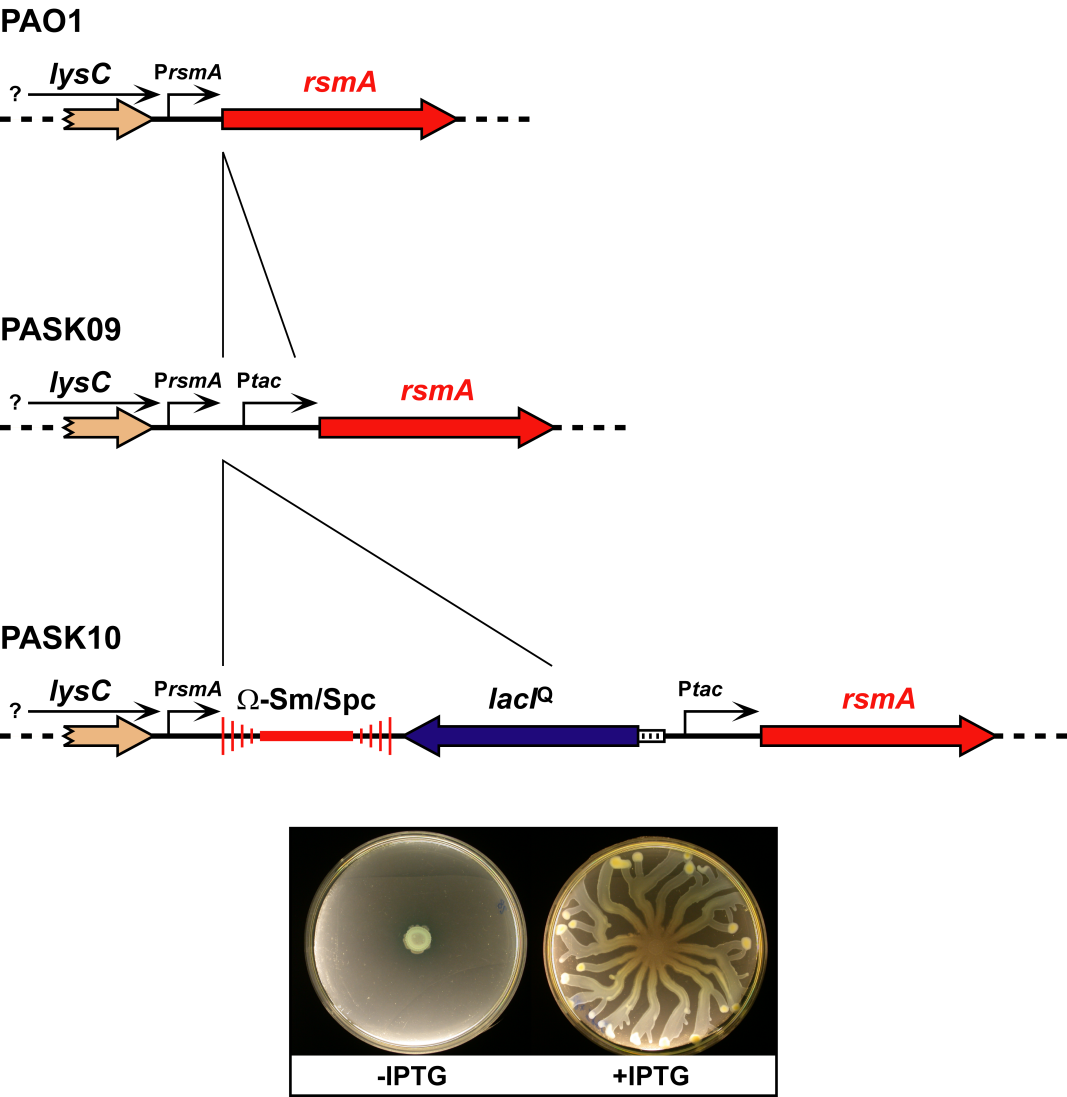


**Supplementary Figure S1**: Construction of *P. aeruginosa* PAO1 derivatives expressing *rsmA* from constitutive or inducible promoters. The *tac* promoter transcribing the *lacZ* 5'UTR and the corresponding ribosome binding site was inserted by allelic exchange immediately upstream of *rsmA* in strain PAO1 to produce strain PASK09, making this gene constitutively overexpressed. In strain PASK10, an omega interposon conferring resistance to streptomycin/spectinomycin was additionally inserted to terminate natural transcription originating upstream of the *tac* promoter, as well as *lacI*^Q^ to achieve transcriptional control. Thus, strain PASK10 behaves like a mutant in the absence of inducer but expresses *rsmA* in the presence of IPTG, as shown by the controllable swarming phenotype.


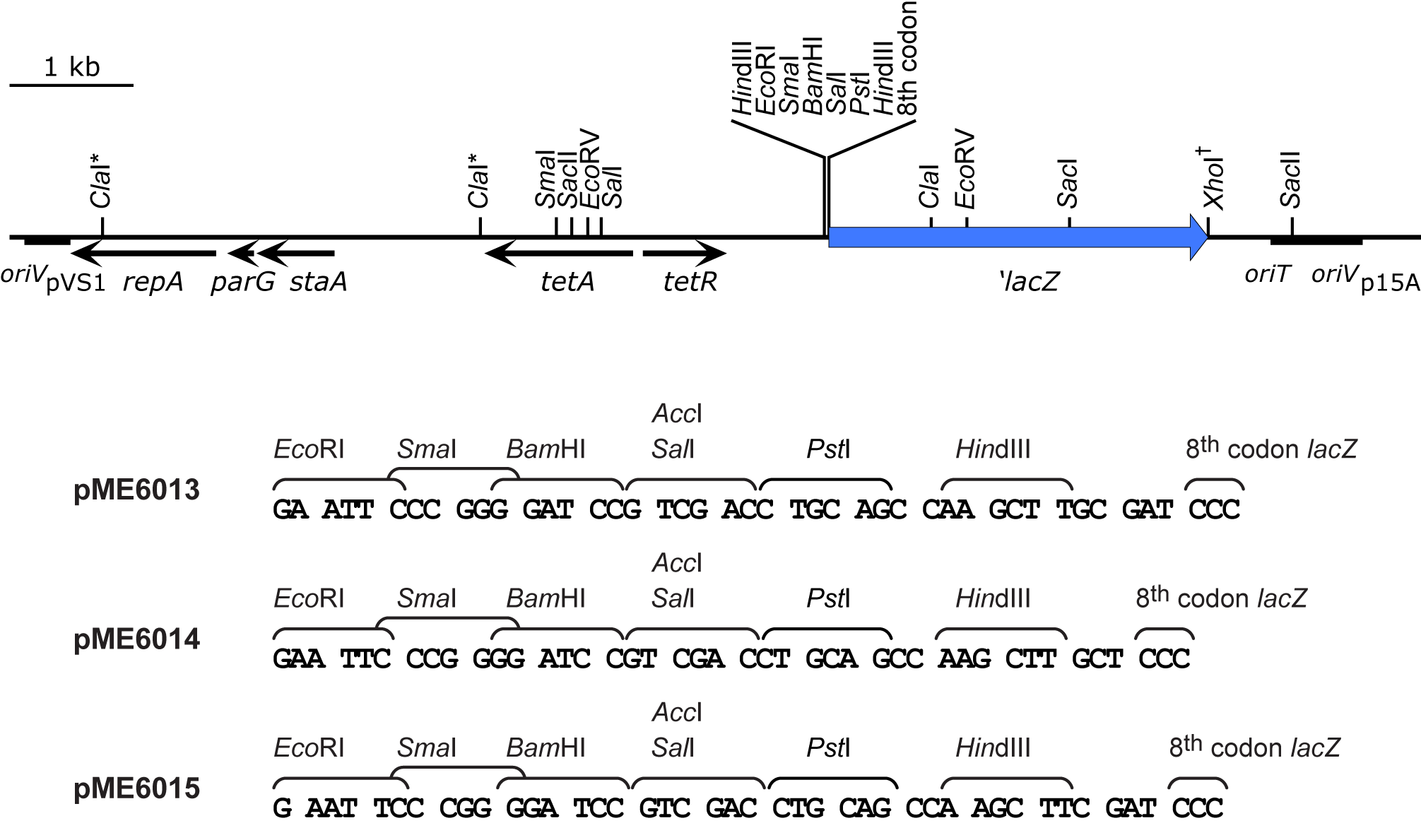


**Supplementary Figure S2**: Construction of the pVS1-p15A shuttle vectors pME6013, pME6014 and pME6013 has been mentioned (90) but not yet fully described. Briefly, these plasmids, suitable for the construction of translational '*lacZ* fusions, were constructed in several steps by subcloning in pME6010 the 3-kb *'lacZ* fragments from, respectively, plasmids pNM480, pNM481 and pNM482 which were engineered to have their multiple cloning sites aligned in all three translational reading frames with *'lacZ* truncated at the 8^th^ codon (Minton 1984, Gene 31:269-273). *Cla*I* sites are methylated in *E. coli* *dam*^+^ strains; due to a different construction strategy, the *Xho*I^†^ site is not present in pME6015.


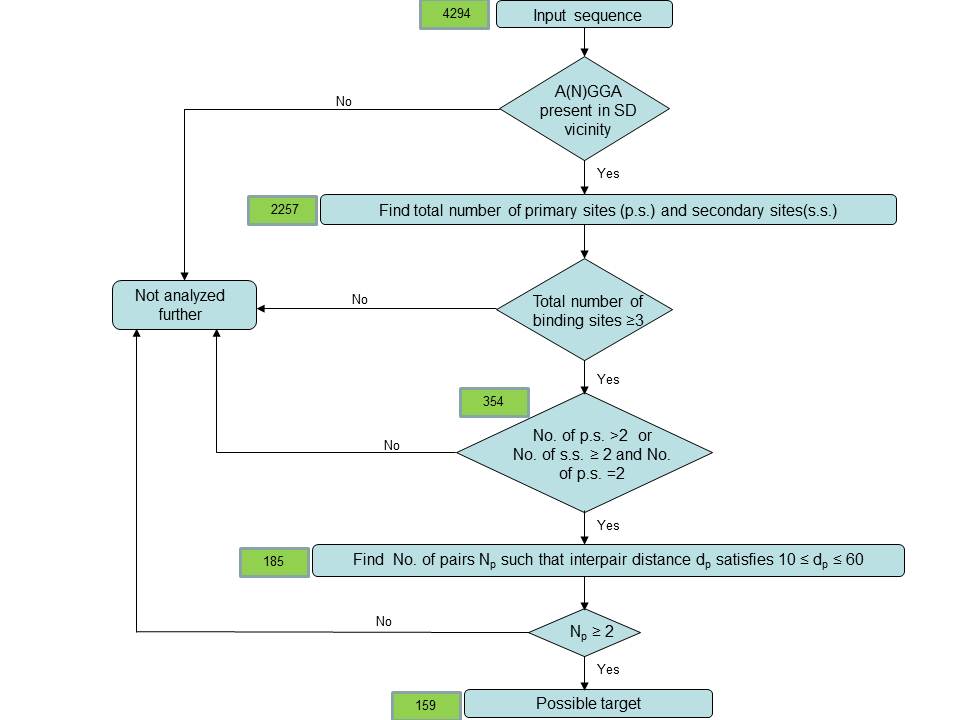


**Supplementary Figure S3**: Flowchart of the algorithm used for the program CSRA_TARGET using *E*. *coli* genome as the sample: The highlighted numbers show the number of target genes that show up after each step.


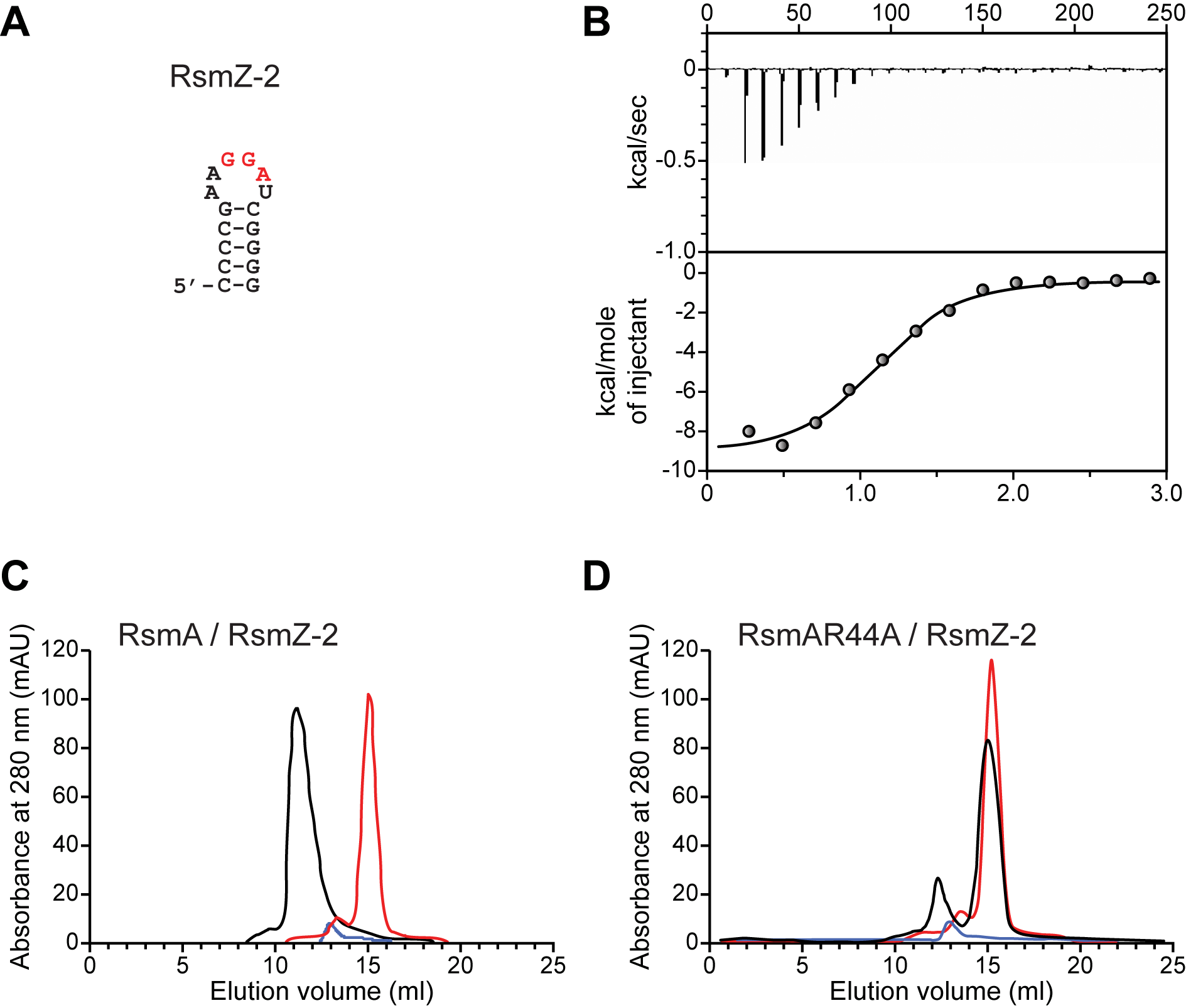


**Supplementary Figure S4**. RsmA/RsmZ-2 binding controls. (**A**) secondary structure of RsmZ hairpin 2 (RsmZ-2) used to verify binding of RNA to RsmA. (**B**) ITC analysis of the binding of RsmZ-2 to the RsmA dimer at 298K (Kd = 276 ±25 nM). 125 μM RNA suspended in 25 mM potassium phosphate buffer, pH 7.0, 50 mM NaCl, was titrated into a cell containing 510 μM protein protein suspended in 1.424 ml of the same solution. The ITC binding isotherms were constructed from the data obtained and fitted to a 1:1 binding model. (**C**) Binding interactions between RsmA and RsmZ-2 determined qualitatively by analytical SEC, showing a shift in retention time of the band for the unbound RNA (red) to faster elution for the complex (black); protein alone is shown in blue. These results show complete conversion of RsmZ-2 to the bound state in the presence of RsmA. (**D**) Same experiment as in (C) but using the substituted protein RsmAR44A. The majority of RsmZ-2 remains in this case unbound in the presence of the protein, indicating a substantially weaker interaction and a much reduced population in the bound state.
